# Regulation of the cohesin-loading factor *NIPBL*: Role of the lncRNA *NIPBL-AS1* and identification of a distal enhancer element

**DOI:** 10.1371/journal.pgen.1007137

**Published:** 2017-12-20

**Authors:** Jessica Zuin, Valentina Casa, Jelena Pozojevic, Petros Kolovos, Mirjam C. G. N. van den Hout, Wilfred F. J. van Ijcken, Ilaria Parenti, Diana Braunholz, Yorann Baron, Erwan Watrin, Frank J. Kaiser, Kerstin S. Wendt

**Affiliations:** 1 Department of Cell Biology, Erasmus MC, Rotterdam, The Netherlands; 2 Section for Functional Genetics at the Institute of Human Genetics, University of Lübeck, Lübeck, Germany; 3 Center for Biomics, Erasmus MC, Rotterdam, The Netherlands; 4 Centre National de la Recherche Scientifique, UMR 6290, Rennes, France; 5 Institut de Génétique et Développement de Rennes, Faculté de Médecine, Rennes, France; Saint Louis University School of Medicine, UNITED STATES

## Abstract

Cohesin is crucial for genome stability, cell division, transcription and chromatin organization. Its functions critically depend on NIPBL, the cohesin-loader protein that is found to be mutated in >60% of the cases of Cornelia de Lange syndrome (CdLS). Other mutations are described in the cohesin subunits SMC1A, RAD21, SMC3 and the HDAC8 protein. In 25–30% of CdLS cases no mutation in the known CdLS genes is detected. Until now, functional elements in the noncoding genome were not characterized in the molecular etiology of CdLS and therefore are excluded from mutation screening, although the impact of such mutations has now been recognized for a wide range of diseases. We have identified different elements of the noncoding genome involved in regulation of the *NIPBL* gene. *NIPBL-AS1* is a long non-coding RNA transcribed upstream and antisense to *NIPBL*. By knockdown and transcription blocking experiments, we could show that not the *NIPBL-AS1* gene product, but its actual transcription is important to regulate *NIPBL* expression levels. This reveals a possibility to boost the transcriptional activity of the *NIPBL* gene by interfering with the *NIPBL-AS1* lncRNA.

Further, we have identified a novel distal enhancer regulating both *NIPBL* and *NIPBL-AS1*. Deletion of the enhancer using CRISPR genome editing in HEK293T cells reduces expression of *NIPBL*, *NIPBL-AS1* as well as genes found to be dysregulated in CdLS.

## Author summary

The most frequent mutations in the human developmental disorder Cornelia de Lange Syndrome (CdLS) occur in the *NIPBL* gene. NIPBL is critical for chromatin-association of the cohesin complex and has a dual role as transcription factor. The regulation of the *NIPBL* gene is of great interest since organisms are very sensitive to NIPBL levels. For instance, severely affected patients showed only ~65% and mildly affected patients ~75% of *NIPBL* mRNA levels. One case reports a CdLS phenotype with as little as 15% reduction of the *NIPBL* transcript. Further, in a number of patients with CdLS phenotype no mutations in known CdLS genes are found, raising the question whether mutations could also occur in gene-regulatory elements. Here we investigate the role of a long non-coding RNA *NIPBL-AS1* originating from a promoter shared with *NIPBL* and observe a co-regulation by a distal enhancer that we have identified and that seems to be conserved between tissues. Deletion of the enhancer by CRISPR leads to reduced expression of *NIPBL* and *NIPBL-AS1* but also of NIPBL target genes that were found to be dysregulated in CdLS patient cells. The lncRNA *NIPBL-AS1* itself has no role for *NIPBL* expression, but its transcription reduces the expression of the *NIPBL* gene and *vice versa*, thus revealing an interesting mechanism for the fine-tuning of expression levels.

## Introduction

Genetic information needs to be inherited without any changes over numerous cell generations and from parents to offspring. This process crucially depends on the cohesin complex that ensures genome stability during cell divisions, DNA damage repair and is involved in the three-dimensional organisation of the chromatin fibre in the cell nucleus [1,2,3]. The association of cohesin with DNA critically depends on the cohesin-loading factor NIPBL [4,5,6]. However, NIPBL is also involved in gene regulation, independent on its role for cohesin [7,8].

*NIPBL* is the gene that is most frequently (>60% of cases, OMIM 122470) found to be mutated in the human developmental disorder Cornelia de Lange syndrome (CdLS, 1 of 10,000–30,000 live births) [9,10] [11,12]. This syndrome is characterized by craniofacial anomalies, upper limb malformations, growth and mental retardation, hirsutism, and other system abnormalities [13,14]. Mutations in the cohesin subunits *SMC1A*, *SMC3*, *RAD21* and the cohesin regulator *HDAC8* [15,16,17,18] account for ~10% of the more moderately affected patients. The genetic causes of 20–25% of CdLS patients are still unknown.

The actual *NIPBL* expression levels seem to be critical for developmental processes in human and mouse. In a cohort of severely affected CdLS patients only ~65% of *NIPBL* expression is observed while mildly affected cases showed ~75% expression; one case report describes a mild CdLS phenotype with as little as 15% reduction of the *NIPBL* transcript [19,20,21]. In *Nipbl* heterozygous knockout mice with a CdLS reminiscent phenotype, the levels of *Nipbl* expression are reduced by only 25–30%, suggesting a compensation by the intact allele [22]. Further, high NIPBL levels have been found to confer poor prognosis in non-small cell lung cancer [23].

In this study we aimed at gaining insight into the regulation of the *NIPBL* gene by identifying regulatory elements in the noncoding genome. Different studies have shown that a large number of potentially disease-causing mutations/variants in the genome are located outside coding regions in regulatory non-coding areas, highlighting the importance of the identification of these elements for the study and diagnosis of human genetic diseases [24] [25] [26]. The potential relevance of such regions for CdLS is illustrated by mutations in the 5’ untranslated region of *NIPBL* (NIPBL:c.-316_-315delinsA; [19] and NIPBL:c.-94C>T; [10]) that affect *NIPBL* expression levels and are disease causing without affecting the NIPBL protein sequence. Gene regulatory elements controlling CdLS genes have not yet been identified and analysed for mutations at all.

During the course of our project, a study aiming at analysing non-coding RNAs overlapping with known autism-related genes identified a *NIPBL* promoter-associated antisense transcript of 5.3 kb, named *NIPBL-AS1*, in brain tissues from patients affected by Autism Spectrum Disorders (ASD) [27]. Since *NIPBL-AS1* and *NIPBL* showed a concordant expression in their hands as well as ours, we hypothesized that *NIPBL-AS1* and *NIPBL* might be interconnected and that this could represent a gene regulatory mechanism.

Long non-coding RNAs (lncRNA) were shown to control genes located in their vicinity in *cis* but also in distant domains in *trans* (reviewed in [28]). These processes can be mediated by the transcription of the lncRNA *per se*, which affects nucleosome positioning or histone modifications at gene promoters [29,30,31,32] or creates a permissive chromatin environment [33]. Alternatively, the lncRNA transcript itself functions as a scaffold to mediate silencing or activation of a target gene by binding to chromatin modifiers, transcription factors or proteins that are part of the transcription preinitiation complex [34,35,36,37,38,39,40]. Thus, we aimed at understanding whether and how the lncRNA *NIPBL-AS1* influences *NIPBL* transcription.

Other crucial gene regulatory elements are enhancers. In mammals, they have been found up to several hundred kilobases from the target transcription start site, as for instance the Sonic Hedgehog (SHH) enhancer that is located 1MB upstream of the gene [41]. Enhancers are “open” chromatin regions marked by H3.3/H2A.Z histone variants and enriched for histone modifications such as mono- and di-methylated lysine 4 of histone H3 (H3K4me1/H3K4me2) and acetylated lysine 27 of histone H3 (H3K27ac) (reviewed in [42]). Enhancers for the *NIPBL* gene were so far unknown. Here we identified an enhancer that stimulates expression of *NIPBL* as well as the lncRNA *NIPBL-AS1*.

## Results

### *NIPBL* gene activity is not influenced by *NIPBL-AS1* transcript

**Fig 1:**
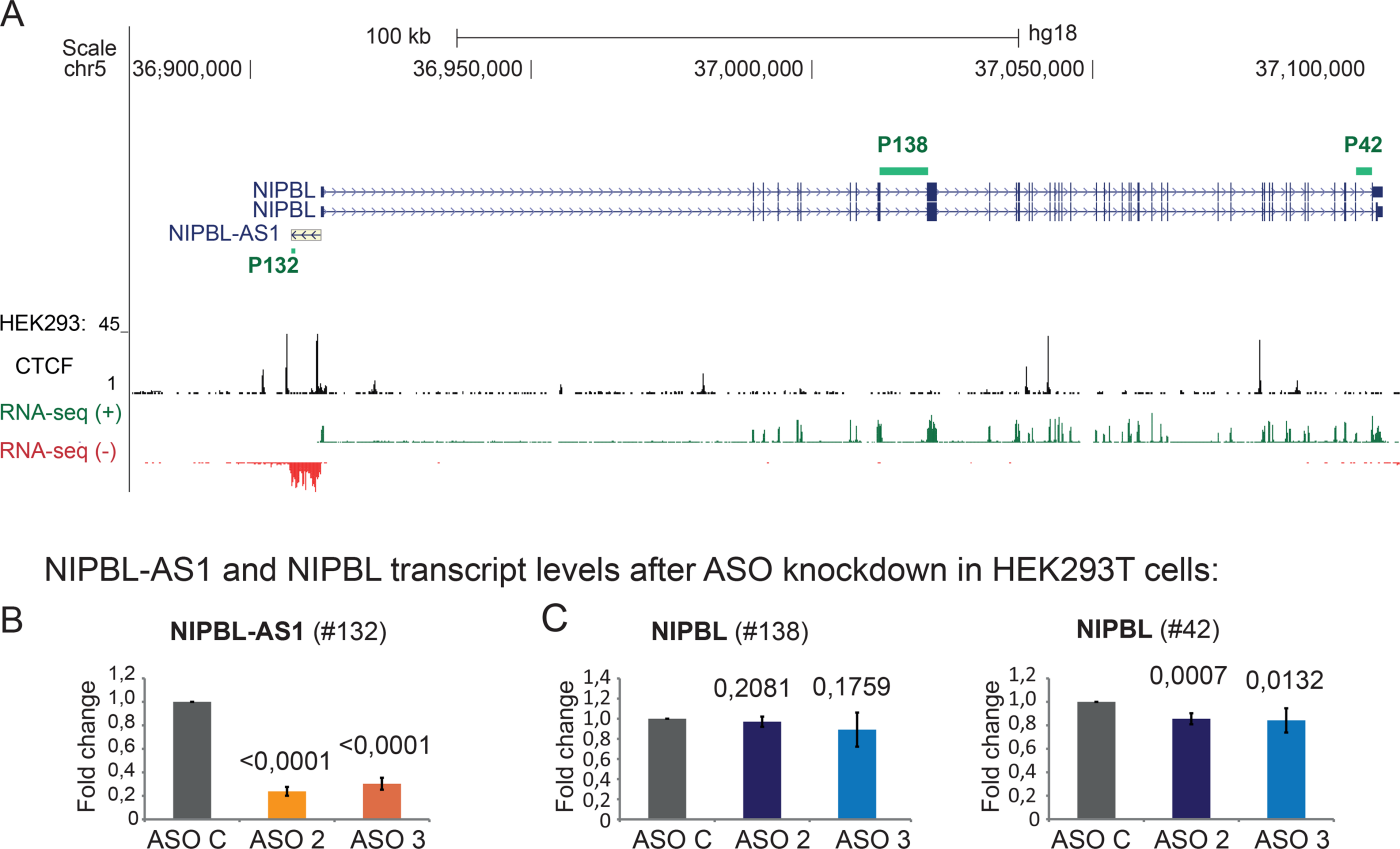
*NIPBL-AS1* does not influence *NIPBL* transcription. A) Overview of the genomic position of *NIPBL* and *NIPBL-AS1* genes. Strand-specific read coverage of RNA-sequencing data (positive in green; negative in red) from HEK293T cells shows the transcription of *NIPBL-AS1* antisense to *NIPBL* [1]. CTCF binding sites in HEK293 cells (ENCODE hg18) are shown. Primers used in the transcript analysis are indicated as green bars. (B-C) Transcript levels of (B) *NIPBL-AS1* and (C) *NIPBL* after antisense oligonucleotide knockdown (ASO2, ASO3) of *NIPBL-AS1* in HEK293T cells. ASO C was used as control. Transcript levels were normalized against the control sample (ASO C) and the housekeeping *SNAPIN* using the ΔΔCt method (mean n = 3, error bars +/- s.d., p-values determined with t-Test).

*NIPBL-AS1* is located upstream of *NIPBL* in a head-to-head orientation and encodes for a 5.3 kb lncRNA (Fig 1A). Considering the emerging roles of lncRNA in genome regulation, we asked whether the *NIPBL-AS1* transcript acts in *NIPBL* expression regulation. Since long noncoding RNAs can function in the nucleus (*MALAT1*, *XIST*) or in the cytoplasm (*linc-MD1*, *NORAD*) [43] we addressed the localization of *NIPBL-AS1* by fractionation of HEK293T cells into cytoplasmic and nucleoplasmic RNA. The RT-qPCR analysis of the cell fractions showed that *NIPBL-AS1* is not retained in the cell nucleus as much as lncRNAs with nuclear functions like *MALAT1* or *XIST* [43]. However a fraction of *NIPBL-AS1* is still present in the nucleus (S1 Fig).

To test whether *NIPBL-AS1* acts directly on *NIPBL* expression, we depleted *NIPBL-AS1* in HEK293T cells using Antisense Oligonucleotides (ASO), which activate the RNAseH pathway by forming DNA/RNA hybrids [44], leading to degradation of the targeted *NIPBL-AS1* RNA. ASOs are in particular suited to knockdown nuclear lncRNA but are also efficient for cytoplasmic lncRNA, so both pools can be efficiently depleted [45,46,47,48]. We used two ASOs to target either the 5’ end or the 3’ end of the *NIPBL-AS1* gene (respectively ASO2 and ASO3) and one non-targeting control (ASO C). The transcript levels of *NIPBL-AS1* (P132) and *NIPBL* (P138; P42) were assessed by RT-PCR/qPCR at 48 hours after transfection. Two intron-spanning primer pairs in the middle (P138) and at the 3’-end (P42) of the *NIPBL* gene that cannot discriminate between the different reported *NIPBL* splice variants [49] were used (Fig 1A). *NIPBL-AS1* transcripts were significantly reduced by both ASOs (Fig 1B), yielding depletion efficiencies for *NIPBL-AS1* as high as 80% for ASO2 and 70% for ASO3. *NIPBL* transcript levels were not affected when we used a primer pair positioned more in the front part of the *NIPBL* gene (primer P138). When we used a primer spanning two of the last exons (P42) we observed a small but significant difference (Fig 1C). To validate our results we repeated our experiment in a different cell line, HB2 cells—a model cell line for normal breast endothelium, that can be easily transfected (S1B and S1C Fig). Here we obtained the same result for primer 138 but the change that we observed for primer 42 is not significant. If the lncRNA would affect *NIPBL* transcription directly we would expect a significant and robust change detectable by both primer pairs. Therefore we concluded that the RNA product originating from *NIPBL-AS1* does not remarkably influence *NIPBL* expression levels.

### Active transcription of *NIPBL-AS1* is important for *NIPBL* transcription

**Fig 2:**
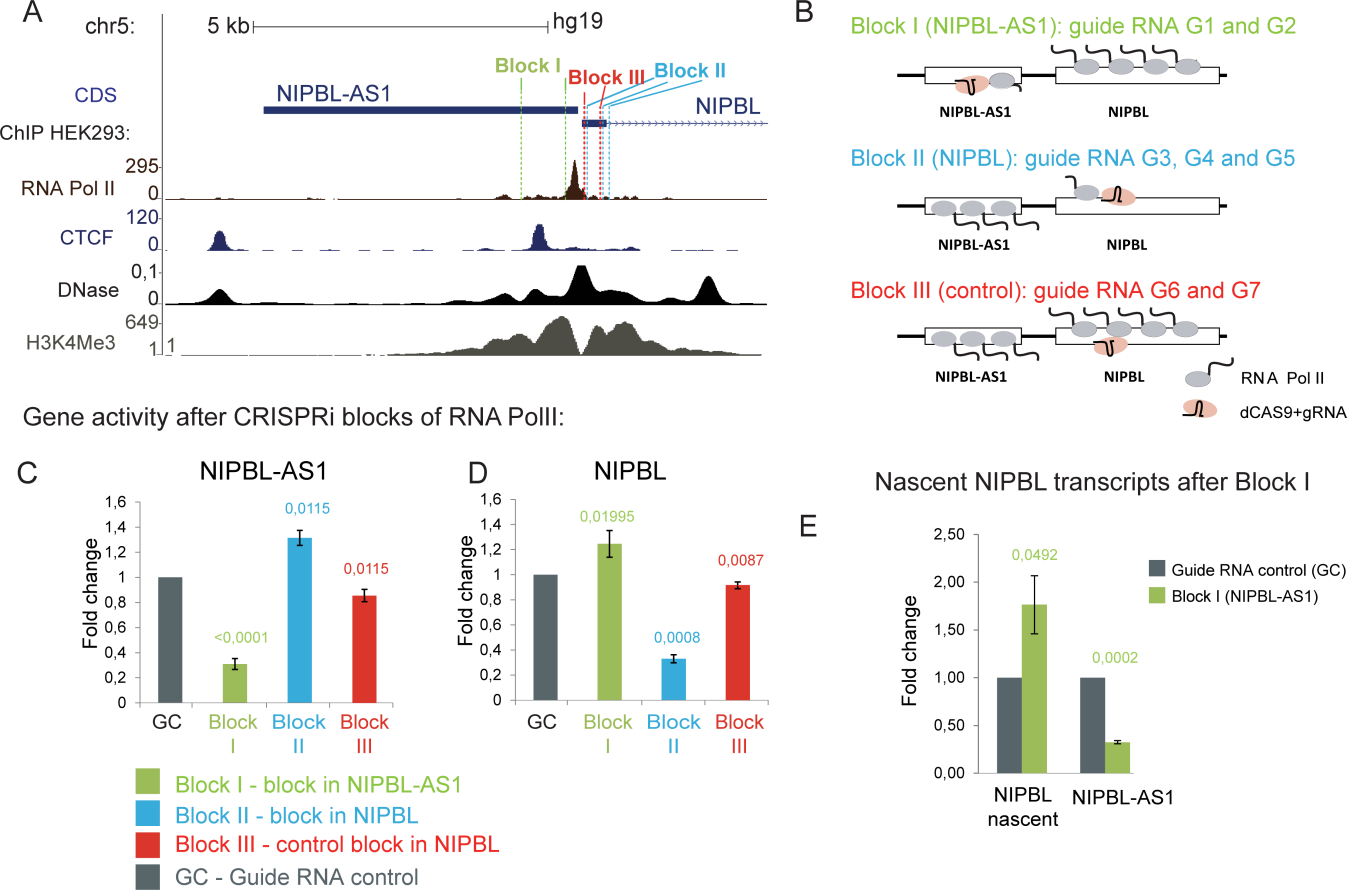
*NIPBL-AS1* and *NIPBL* are transcribed from a shared bi-directional promoter. A) Overview of the *NIPBL-AS1* and the *NIPBL* promoter region together with ChIP-seq data for RNA polymerase II, CTCF, the H3K4me3 histone mark and DNase hypersensitive regions in HEK293 cells (ENCODE). The locations of the different guide RNAs used for the CRISPRi blocks (Block I, Block II and Block III) are shown. B) Overview scheme of the CRISPRi blocks to interfere with transcription of *NIPBL-AS1* (Block I), *NIPBL* (Block II) and a non-interfering control block (Block III). Note that the interfering gRNAs for block I and block II were designed complementary to the non-template DNA strand and for block III complementary to the template strand [50]. C-D) Transcript levels of *NIPBL-AS1* and *NIPBL* under the different blocks normalized to a control transfected with a guide RNA positioned in an intergenic region on chr5 unrelated to *NIPBL* (GC). Note that block III does not interfere with the transcription of *NIPBL-AS1* and *NIPBL*. (C) *NIPBL-AS1* levels are reduced under block I targeting *NIPBL-AS1* but increased under block II targeting *NIPBL*. (D) *NIPBL* levels are reduced under block II targeting *NIPBL* but increased under block I targeting *NIPBL-AS1* (mean of n = 6; error bars +- s.d., p-values determined with t-Test). (E) The level of the nascent *NIPBL* transcript was determined when *NIPBL-AS1* transcription was blocked (Block I) to determine whether the *NIPBL* upregulation seen in (D) originates from de-novo transcription.

*NIPBL* and *NIPBL-AS1* are transcribed from a shared bidirectional promoter that is GC rich and lacks TATA elements. The annotated transcription start sites (TSS) of both genes are separated by 77 bp (Fig 2A). The promoter is characterized by a DNAse hypersensitive region flanked by mirrored H3K4me3 marks (Fig 2A). Only little is known about the regulation of bidirectional promoters and about the relationship between the two transcribed genes. Since the *NIPBL-AS1* transcript seems to have no relevant function for *NIPBL* regulation, we hypothesized that the actual transcription of *NIPBL-AS1* is important for *NIPBL* expression.

To investigate this, we used the CRISPR/Cas9 system to interfere with the transcription process of *NIPBL-AS1* and *NIPBL* using different specific guide RNA (CRISPRi) (Fig 2A and 2B). It has already been reported that the DNA-binding of the rather bulky complex of catalytically inactive Cas9 (dCas9) and guide RNA (gRNA) is sufficiently stable to block the progression of RNA polymerase II, leading to silencing of the target gene [50]. We designed gRNA for three different dCAS9 blocks (Block I- targeting *NIPBL-AS1*, Block II—targeting *NIPBL*, Block III—placed in the *NIPBL* gene but not blocking) as well as a control gRNA (GC) in distant gene desert on chromosome 5. Since it was shown that gRNAs targeting the non-template DNA strand have a higher gene silencing effect compared to those targeting the template strand [50], we recruited dCas9 to the 5’ end of *NIPBL-AS1* using two guide RNAs (Block I, G1 and G2) targeting the non-template strand of *NIPBL-AS1* (Block I, Fig 2A and 2B). To block transcription of *NIPBL*, we used three guide RNAs (G3, G4 and G5 –Block II) targeting the non-template strand of the gene (Block II, Fig 2A and 2B). To verify the specificity of the transcription block we used two gRNAs (Block III, G6 and G7) designed complementary to the template strand of *NIPBL* (Block III, Fig 2A and 2B) since gRNAs targeting the template strand do not efficiently block gene transcription. The gRNAs for each block were mixed and co-transfected with a construct coding for catalytically inactive Cas9 (dCas9) into HEK293T cells.

First, we analysed the wider promoter region of *NIPBL-AS1* and *NIPBL* by ChIP-qPCR (for primer positions see S2A Fig) for the coverage of the initiating Polymerase II, marked by phosphorylation of the C-terminal Ser5 residue (RNA PolII Ser5) [51,52,53,54], and the total RNA Polymerase II (RNA Pol II). We observed an increased accumulation of RNA PolII Ser5 upstream of the guide RNA target sites of Block I compared to the control block (Block III) (see S2B Fig, primer AS3). Vice versa we observed in Block II a similar accumulation of RNA PolII Ser5 upstream of the guide RNA target sites (S2D Fig, primer AS7). For both blocks we also observed a slight increase in total RNA Pol II signals upstream of the guide RNA target sites (S2C Fig, primer AS3 and S2E Fig, primer AS7). RNA PolII and RNA PolII Ser5 signal increments tend to spread on the respective gene that was not blocked (*NIPBL* for Block I and *NIPBL-AS1* for Block II).

We then analysed the transcript levels of *NIPBL* and *NIPBL-AS1* under the different blocks. When we transfected the cells with Block I, only 30% of the *NIPBL-AS1* transcript was still detectable compared to the control guide RNA transfection (GC), indicating that *NIPBL-AS1* transcription was significantly blocked (see Block I, Fig 2C). *NIPBL* mRNA levels after blockage of *NIPBL-AS1* transcription were increased (Fig 2D). By using a primer pair detecting the unspliced *NIPBL* transcript (Fig 2E) we confirmed that the increase in *NIPBL* transcripts originates from *de-novo* transcription and not from increased stability of the mRNA. In cells transfected with Block II we could achieve a significant reduction of *NIPBL* transcription, with only 30% of the transcript still detectable (Fig 2D). We observe now an upregulation of the *NIPBL-AS1* transcript (Fig 2C), similar to the observations for the *NIPBL* transcript in Block I. In cells transfected with Block III we did not observe a relevant reduction of *NIPBL* or *NIPBL-AS1* transcription (Fig 2C and 2D).

In summary, we could achieve an efficient block of the transcription of *NIPBL* and *NIPBL-AS1* (Fig 2). This is probably due to the accumulation of RNA PolII and RNA PolII Ser5 at the block sites (see primer AS3 and AS7 in S2 Fig). The accumulation of PolII Ser5 is more evident than that of RNA PolII; this is consistent with the presence of a stalled RNA PolII, since RNA PolII Ser 5 is also a mark for stalling. [51,52,53,54]. This leads to a reduced expression of the gene involved in the blocking but also a significant upregulation of the corresponding gene that is not blocked. We have therefore demonstrated that the transcription of *NIPBL-AS1* and *NIPBL* is indeed interconnected and that by reducing the transcription activity of *NIPBL-AS1* we increased the transcription at the *NIPBL* gene and *vice versa*. We have also uncovered an interesting approach to eventually manipulate the expression of the *NIPBL* gene without interfering with the *NIPBL* gene itself.

### Identification of a distal enhancer for the *NIPBL* gene

**Fig 3:**
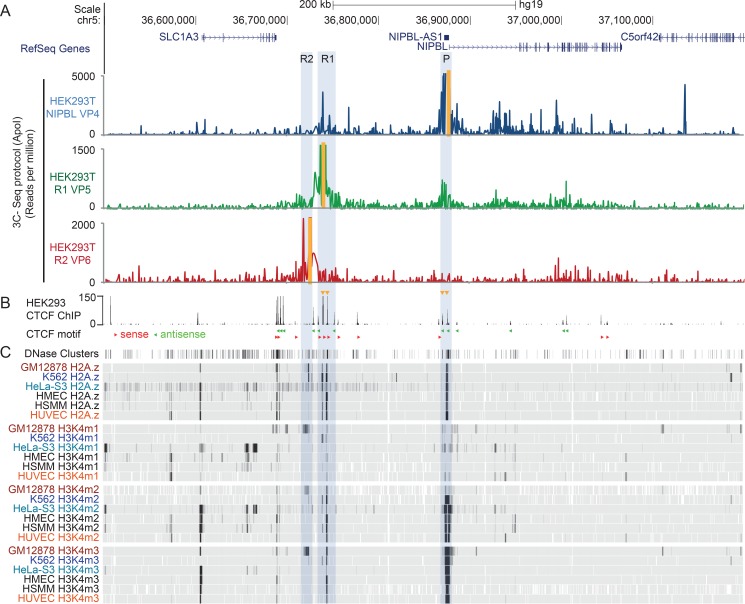
Interactions of *NIPBL* and *NIPBL-AS1* with a potential distal enhancer. A) Long-range chromosomal interactions of the *NIPBL* and *NIPBL-AS1* promoter detected by chromosome conformation capture (3C-seq) in HEK293T cells using an ApoI digest. The positions of the different viewpoints used are marked in yellow. Three different viewpoints at the promoter (VP4, blue track) and the candidate enhancers regions R1 (VP5, green track) and R2 (R2—VP6, red track) were used. B) CTCF ChIP sequencing track from HEK293 cells (ENCODE). The orientations of the CTCF motifs as determined with JASPAR are shown below the track (red triangle–forward orientation, green triangle–reverse orientation). The CTCF sites involved in the promoter-enhancer interaction are indicated with yellow triangles above the track. C) DNAse clusters as well as histone modification profiles—H2A.z, H3K4me1, H3K4me2 and H3K4me3—of six different cell lines (G312878, K562, HeLa-S3, HEMEC, HSMM and HUVEC, available from ENCODE) are displayed as density graph. Black represents areas with the highest enrichment of the signals.

We aimed at identifying the distal regulatory elements of *NIPBL* and *NIPBL-AS1* using chromosome conformation capturing (3C-seq, a derivative of 4C) [55], initially in the HB2 cell line but also in HEK293T cells since these cells are better suitable for CRISPR genome editing. Using BglII as 3C-seq restriction enzyme and a viewpoint located at the *NIPBL* promoter (VP1), we observed contacts to two intergenic regions, namely R1 and R2, respectively at 130 kb and 160 kb upstream of the *NIPBL* promoter (S3A Fig). We focused on these regions since the analysis of ChIP sequencing data for different histone marks and DNaseI hypersensitivity tracks for six cell lines (GM1287, K562, HeLa-S3, HMEC, HUVEC, HSMM), available from the ENCODE project [56], revealed that the R1 region highly correlates with open chromatin (DNAseI) and histone variants/marks found at enhancers and active transcription (H2A.Z, H3K4me1, H3K4me2, H3K4me3) [57] in different cell lines (Fig 3C, S3C and S3D Fig). Region R2 correlates only in GM12878 cells with enhancer marks. Thus, we hypothesized that R1 is the best candidate for a *NIPBL* distal enhancer. The 3C-seq analysis also revealed contacts from the *NIPBL* promoter into the *NIPBL* gene body, covering nearly all of the 47 exons (188 kb), also confirmed by two additional viewpoints inside *NIPBL* (VP2-3) (S3A Fig). However, since these contacts do not involve regions with characteristic enhancer marks we omitted them from our search for *NIPBL/NIPBL-AS1* enhancers.

To confirm the observed long-range interactions of the *NIPBL* promoter, we performed a higher resolution 3C-seq (using ApoI as restriction enzyme) using one viewpoint at the *NIPBL* promoter (VP4) and two viewpoints at R1 (VP5) and R2 (VP6) in HEK293T cells (Fig 3A) and HB2 cells (S3B Fig). These experiments confirmed the contact between the *NIPBL* promoter and the distal intragenic region R1 (Fig 3A, S3B Fig, blue tracks, VP4). *Vice versa*, the viewpoint located at R1 (VP5) showed contacts between this potential enhancer and the promoter of *NIPBL* and *NIPBL-AS1* (or 5’end of *NIPBL-AS1*) (Fig 3A, S3B Fig, green tracks, VP5). These interactions involved two CTCF binding sites at R1 and two within *NIPBL-AS1* respectively (see CTCF ChIP-sequencing peaks in HEK293 cells in Fig 3B or S3C Fig), that are positioned in the forward-reverse motif orientation that favours long-range interactions (Fig 3B) [58,59,60]. The interaction between R2 (VP6) and the *NIPBL* promoter is no longer observed (Fig 3A, S3B Fig, red tracks, VP6). Therefore we consider from now on only R1 as candidate enhancer.

Consistent with our observations, recently published high resolution Hi-C data showed contacts between the *NIPBL* promoter and the distal region R1 in seven different human cell lines (GM12878, HMEC, NHEK, KBM7, HUVEC, K562, IMR90) [59] (S4A and S4G Fig). This indicates conservation of this long-range interaction between different cell types, eventually also between species since Hi-C data from mouse CH12 cells also showed long-range contacts of the *NIPBL* promoter to a potential distal enhancer next to the *Slc1a3* gene (S4H Fig).

### Functional characterization of the candidate enhancers

**Fig 4:**
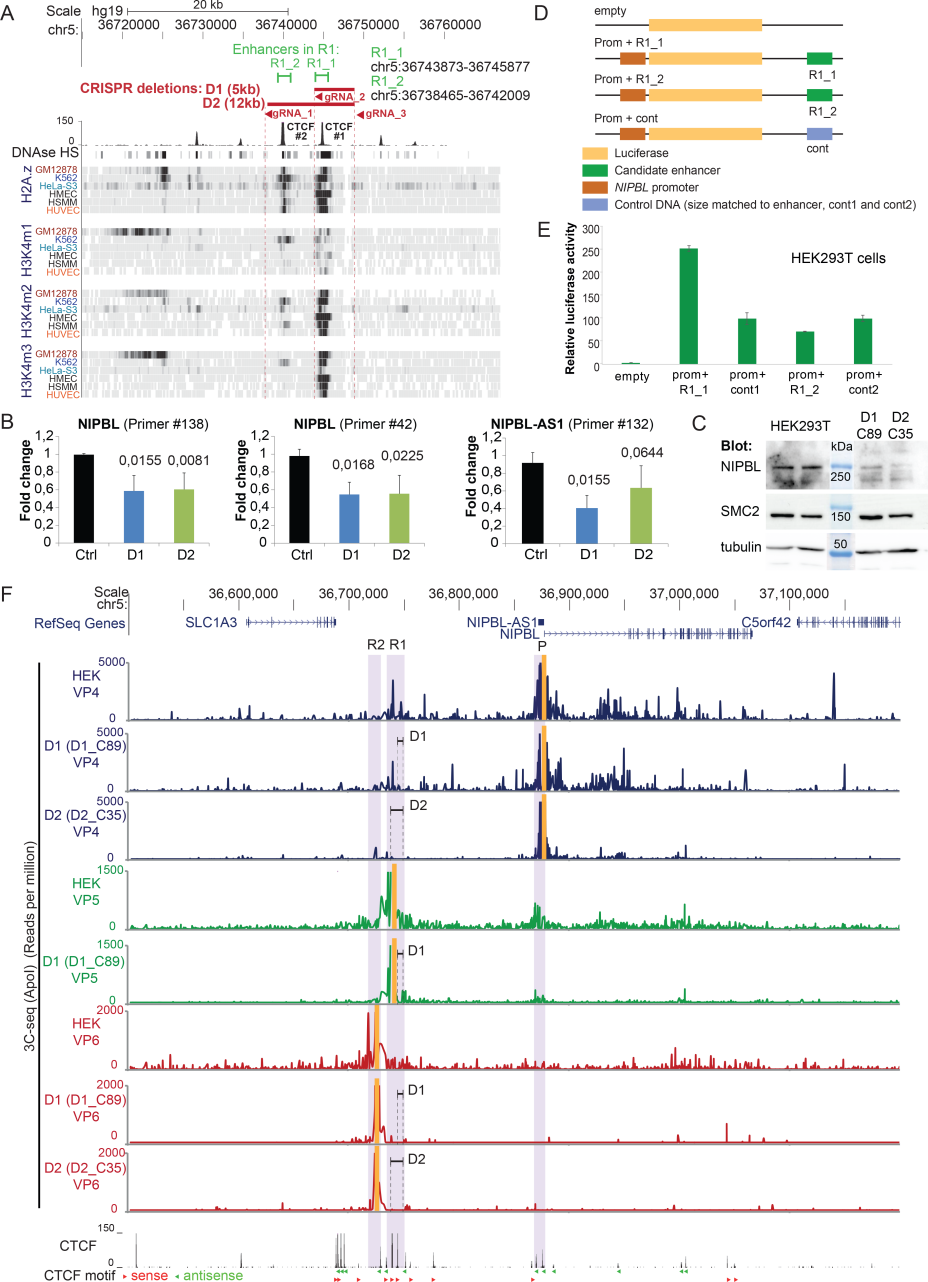
Deletion of the candidate enhancers by CRISPR. A) The candidate enhancer region R1 contains two regions with characteristic histone marks termed R1_1 and R1_2. CRIPSR genome editing was used to delete R1_1 (D1, gRNA_2 and gRNA_3) and R1_1 and R1_2 together (D2, gRNA_1 and gRNA_3). B) Average transcript levels of *NIPBL* and *NIPBL-AS1* in the clones with the candidate enhancer regions R1_1 deleted (D1) and R1_1 together with R1_2 (D2) deleted by CRISPR editing in HEK293T cells. The transcript levels of the individual clones are shown in S6 Fig. Two primers within *NIPBL* and one located at the 3’ end of *NIPBL-AS1* were used. Transcript levels were normalized against the housekeeping gene *SNAPIN* (mean n = 5 for D1 and n = 4 for D2, error bars +/- s.d., p-values determined with t-Test). C) Detection of NIPBL protein levels in two enhancer deletion clones (D1_C89 and C_35) by western blotting. The condensin subunit SMC2 and tubulin were used as loading controls. D) Cloning scheme of the luciferase assay constructs with the candidate enhancers R1_1 and R1_2. The luciferase gene is expressed under the control of the *NIPBL* promoter. The candidate enhancer or an equally sized control sequence is positioned at the opposite side of the gene. E) Relative luciferase activity obtained from the different constructs after transfection of HEK293T cells (mean of n = 3; error bars +- s.d.). F) Long range-interactions of the *NIPBL* and *NIPBL-AS1* promoter region after deletion of R1_1 (D1, clone D1_C89) and deletion or R1_1 and R1_2 (D2, clone D2_C35) of the potential enhancer in HEK293T cells. The same viewpoints as in Fig 3 are used located at the promoter region (VP4) and in the candidate enhancer regions R1 (VP5) and R2 (VP6), the position of the viewpoints is highlighted in yellow. Positions and the size of the deletions are indicated. Below the 3C-seq experiments the CTCF sites from HEK293 cells (ENCODE) are shown with the orientations of the CTCF motifs indicated (red triangle–forward orientation, green triangle–reverse orientation).

Since we identified R1 as potential distal regulatory element, we wanted to test whether this element displays activity typical for enhancers with respect to *NIPBL* and *NIPBL-AS1*. For this we deleted R1 using CRISPR/Cas9 genome editing in HEK293T cells. The R1 region comprises two fragments, which we termed R1_1 and R1_2, enriched in histone marks typical for enhancers and actively transcribed regions as well as CTCF sites (CTCF#1 in R1_1 and CTCF#2 in R1_2) (Fig 4A). To dissect the contribution of R1_1 and R1_2 in the regulation of *NIPBL* and *NIPBL*-*AS1* we designed guide RNA that specifically delete either a region including R1_1 (D1, gRNA2 and gRNA3) or R1_1 and R1_2 at the same time (D2, gRNA1 and gRNA3) (Fig 4A). In total we obtained four clones (D1_89, D1_38, D1_42, D1_63) for the smaller deletion of 5 kb (D1) and five clones (D2_35, D2_18, D2_25, D2_33, D2_103) for the 12 kb deletion (D2). The homozygous targeting was confirmed by PCR where we obtained an amplification only for primers designed to detect the deletion, but not for primers designed to detect the intact genomic region (see S5D and S5H Fig).

We assessed the effects of these two deletions on the expression of *NIPBL*. RT-qPCR analysis showed a reduction of *NIPBL* transcript levels to in average 60% for both depletions and transcription of *NIPBL-AS1* was also reduced (Fig 4B, S6 Fig). The reduction of NIPBL expression can also be detected at the protein level by western blotting (Fig 4C). This supports that R1 acts as distal enhancer of *NIPBL*. We concluded that the actual enhancer localizes in the R1_1 fragment since the larger deletion including also R1_2 does not lead to more significant changes in *NIPBL* and *NIPBL-AS1* transcript levels.

In order to validate the enhancer activity of R1_1 and R1_2 in an independent experimental setup, we cloned the two fragments in plasmids carrying a luciferase gene under control of the *NIPBL* promoter (Fig 4D). Two similar sized random DNA fragments were used as controls (Fig 4D, cont1, cont2). The constructs were transfected into HEK293T cells and the luciferase activity analysed. In comparison to the control, DNA fragment R1_1 leads to a clear 2.5 fold increase of the luciferase activity but not R1_2 (Fig 4E). Taken together, these results point to R1_1 as the enhancer for *NIPBL* and *NIPBL-AS1*.

To investigate how the deletions D1 (including R1_1) and D2 (including R1_1 and R1_2) affect the long-range interactions of the *NIPBL* promoter, we performed 3C-seq with one clone from D1 (D1_C89) and one clone from D2 (D2_C35) (Fig 4F) using the *NIPBL* promoter (VP4), R1 (VP5) and R2 (VP6) as viewpoints (Fig 4F). The D1 deletion removed the CTCF#1 site (S2A Fig and Fig 4D) but the promoter remains in contact with the CTCF#2 site (see Fig 4F, VP4 and VP5 panels for D1). When both CTCF sites (CTCF#1 and CTCF#2) are deleted (D2) only very little interactions of the promoter region (Fig 4F, VP4 panel for D2) into the area surrounding the deleted enhancer regions are detectable, as well as the contacts into the *NIPBL* gene body.

We conclude that the *NIPBL/NIPBL-AS1* enhancer overlaps with the R1_1 region but both CTCF sites in R1 (CTCF#1 and CTCF#2) are required for the long-range interactions between R1 and *NIPBL/NIPBL-AS1*.

### Relevance for Cornelia de Lange Syndrome (CdLS)

**Fig 5:**
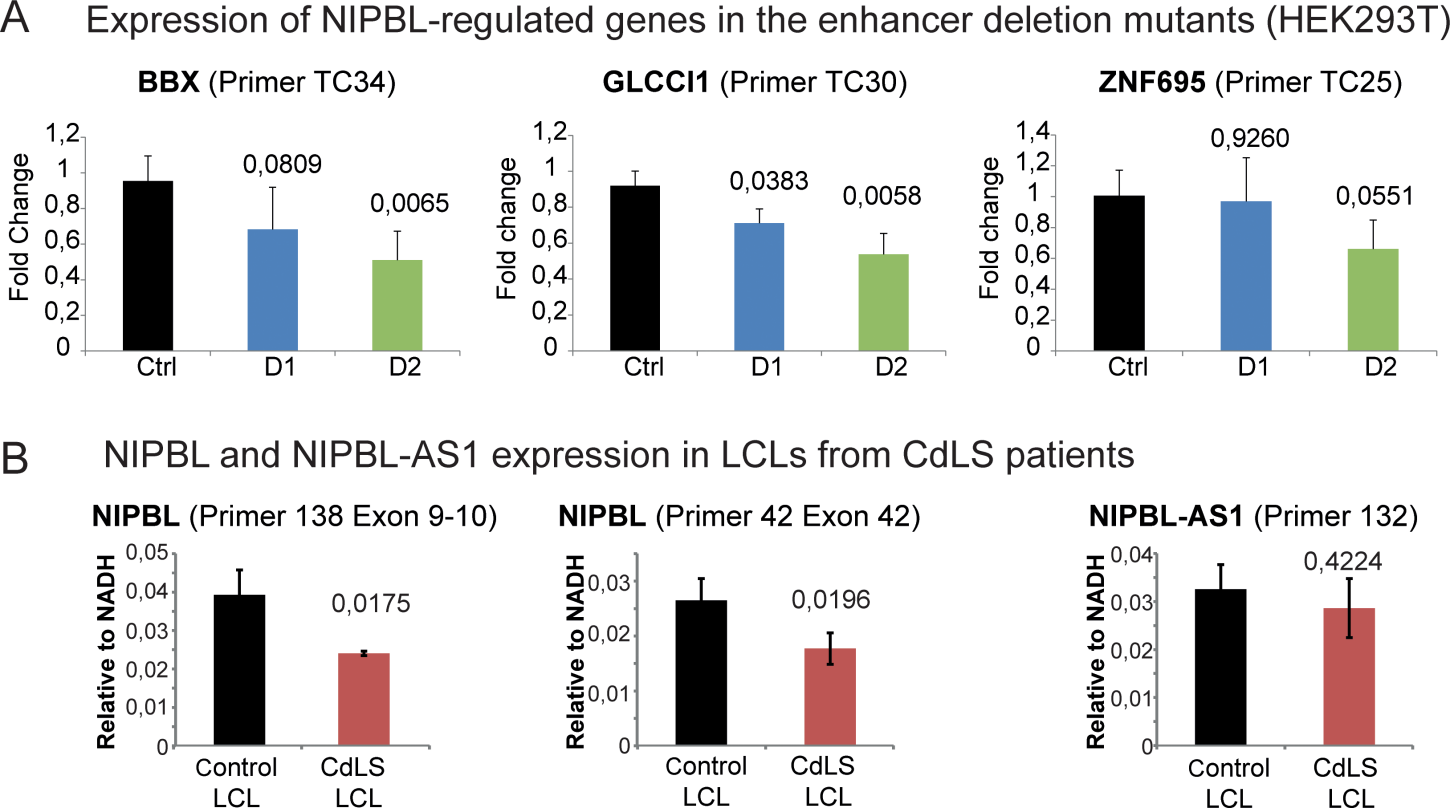
Implications for CdLS. A) Transcript levels of the genes *BBX*, *GLCCI1* and *ZNF695* that were described as dysregulated genes in CdLS [20] and previously confirmed as NIPBL-dependent genes with NIPBL binding sites at the promoter [8] were analysed in the different enhancer deletion clones D1 and D2 (mean n = 5 for D1 and n = 4 for D2, error bars +/- s.d., p-values determined with t-Test, the transcript levels of the individual clones are shown in S7 Fig). B) Average transcript levels of *NIPBL* and *NIPBL-AS1* in lymphoblastoid cell lines (LCLs) derived from CdLS patients and controls. The details of the four LCL controls and three CdLS LCLs as well as the individual transcript levels are shown in S8 Fig and in [8,20]. Two primer pairs for *NIPBL* and one for *NIPBL-AS1* were used. Transcript levels were normalized against the housekeeping gene *NADH* (mean n = 4 for control LCLs and n = 3 for CdLS LCLs, error bars +/- s.d., p-values determined with t-Test).

To demonstrate the importance of the R1_1 enhancer we asked whether the deletion of the enhancer affects genes that were found to be dysregulated in CdLS patients [20] and that we previously confirmed to be regulated by NIPBL [8]. RT-qPCR analyses revealed that all three analysed genes (*BBX*, *ZNF695*, *GLCCI1*) are downregulated in the larger deletion (D2) and two genes in the smaller deletion (D1) (Fig 5A and S7 Fig). This further supports the relevance of the identified enhancer region for the regulation of *NIPBL* and also of the NIPBL downstream targets which are dysregulated in CdLS.

*NIPBL* transcript levels were found to be reduced in CdLS patients with *NIPBL* mutations but the cause of this downregulation was unclear [8,20]. We were curious to check *NIPBL* and *NIPBL-AS1* transcript levels in cells from CdLS patients with a heterozygous truncation mutation of *NIPBL* [20,21]. We assessed levels of *NIPBL* transcripts in three patient LCLs with early truncating mutations in *NIPBL* (PT1-3) as well as in four control LCLs (C1-4) (described in [8,20] and S8A Fig). Consistent with previous reports [20,21], the *NIPBL* mRNA levels in the patient cells were reduced to 60–70% (Fig 5B and S8B Fig). Explanations for this imply either a downregulation of the *NIPBL* gene and/or a degradation of the *NIPBL* transcript by the nonsense-mediated mRNA degradation pathway, as has been previously hypothesized [61]. In the first case we might expect a misregulation of *NIPBL-AS1* since our data show that the transcription of *NIPBL* and *NIPBL-AS1* are interconnected. In the second case we would not observe an effect on *NIPBL-AS1*. Assessing the *NIPBL-AS1* in the patient cells showed that the transcript levels of *NIPBL-AS1* were not significantly affected (Fig 5B, S8B Fig). Next we analysed by pyrosequencing the fraction of the mRNA originating from *wt* and mutant alleles. Here we observed 38% (PT1) or 24% (PT3) of mutant transcripts (S8C Fig) which increase up to 46–48% when blocking nonsense-mediated mRNA decay by cycloheximide, indicating at least a partial degradation of mutant *NIPBL* transcripts in patient cells, while both alleles remain actively transcribed. This explains why *NIPBL-AS1* transcription is not altered and supports our finding that the *NIPBL-AS1* transcript is not involved in *NIPBL* transcription and vice versa (Fig 1).

## Discussion

*NIPBL* encodes for a protein that is required to load the cohesin complex onto DNA but has also a role as transcription factor [8]. It is also the most frequently mutated gene in Cornelia de Lange syndrome (>60% of the cases). However, in about 25–30% of the cases the genetic cause of the disease is unknown. Some of these cases might be explained by mosaicism [62,63], but the disease-causing mutations might also reside in gene-regulatory regions that are unknown and therefore not covered in diagnostics.

Interestingly, in heterozygous *Nipbl* knock-out mouse tissues the *Nipbl* transcript was found to be only reduced by 25–30% and a compensatory regulatory mechanism for *Nipbl* was suggested [22]. Therefore, the gene regulation of *NIPBL* is of great interest for eventual approaches to manipulate the expression of the gene.

Here we characterized in depth the regulation of the *NIPBL* gene by a bidirectional promoter controlling the transcription of *NIPBL* and of the 5.3 kb lncRNA *NIPBL-AS1*. We investigated the role of the lncRNA for *NIPBL* expression and identified and characterized a conserved distal enhancer that stimulates *NIPBL* and *NIPBL-AS1* expression.

It has been shown that lncRNAs can function either through their RNA product or through their active transcription. To test this systematically we first depleted *NIPBL-AS1* by antisense oligonucleotides (ASO) without observing a significant change in *NIPBL* expression (Fig 1B and 1C). Similarly, reduction of the *NIPBL* transcript in CdLS patient LCLs with heterozygous NIPBL truncation mutations, at least in part due to nonsense-mediated mRNA decay, does not alter the level of lncRNA transcription (Fig 5B and S8 Fig). Therefore the RNA products of both genes seem to do not influence each other. This is consistent with the observation that the *NIPBL-AS1* sequence is not conserved among mammals.

To test whether the transcription of the lncRNA from the bidirectional promoter is important for *NIPBL* expression, we blocked the transcription of *NIPBL-AS1* using CRISPRi [50] and observed an upregulation of the *NIPBL* transcript. Vice versa, blockage of the transcription of *NIPBL* lead to an upregulation of the lncRNA (Fig 2C and 2D). This underlines an interesting mechanism that could be potentially explored for the manipulation of *NIPBL* expression levels in clinical applications.

Antisense transcription has been observed for a large number of promoters in the human genome [64,65,66]. The *NIPBL-AS1* is a rather long unspliced noncoding RNA (5.3 kb), transcribed from a bidirectional promoter shared with *NIPBL*. Both genes seem to be transcribed to comparable levels according to the RNA-seq signals (Fig 1A) and our transcript analysis in LCLs (Fig 5B). How the transcription at such bidirectional promoters is regulated is still unclear (for a review see [67]); therefore our observation that the transcripts are coupled in such manner that blocking the transcription of one gene leads to increased transcription of the other gene may represent a novel mechanism in fine tuning of gene expression.

We also identified one distal enhancer of *NIPBL* and *NIPBL-AS1* located 130 kb upstream of the promoter within a region that we call R1 (Fig 3). This region contains two CTCF sites: the one facing the *NIPBL* gene (CTCF #1) correlates strongly with active chromatin marks (e.g. H3K4me1, H3K4me2, H3K4me3) while the second one (CTCF #2) shows only little correlation with those histone marks (Fig 4A). The deletion of 5 kb (D1) including a region (R1_1) enriched in enhancer marks and including the CTCF#1 site led to downregulation of both *NIPBL* and *NIPBL-AS1* expression. A larger deletion of 12 kb (D2, comprising R1_1 and R1_2) that included CTCF#1 and CTCF#2 did not lead to further downregulation of expression. The enhancer of NIPBL/NIPBL-AS1 resides therefore in R1_1 and overlaps with CTCF#1.

Interestingly, the 3C-seq experiments reveal that two CTCF binding sites close to *NIPBL/NIPBL-AS1* bidirectional promoter strongly contact the two CTCF binding sites within the R1 region. The motif orientation of these sites is consistent with the preferential motif orientation for loop formation. Both sites in R1 seem to be required for the long-range interactions of the *NIPBL/NIPBL-AS1* promoter. This promoter-enhancer interaction seems to be conserved between different tissues and also across species (S4A and S4H Fig). We propose that the interactions between these CTCF sites are important for the recruitment of the enhancer to the *NIPBL/NIPBL-AS1* promoter. It remains to be shown whether this enhancer-promoter loop is a permanent loop scaffold, as suggested for developmental genes in fly [68] and TNF-α-responsive genes in human [69]. Due to the close distance of the two TSS, the looped enhancer might stimulate alternatively the transcription at the TSS of *NIPBL* or *NIPBL-AS1*, suggesting a competition between both genes for the enhancer activity. This is supported by our observation that blocking the transcription of one gene, with RNA Pol II Ser5 accumulating at the block site, leads to increased transcription of the other gene.

An interesting observation concerning the *NIPBL* gene are the intensive contacts of the *NIPBL* promoter all over the *NIPBL* gene covering nearly all of the 47 exons (188 kb) (S3 Fig). Since the *NIPBL* intragenic viewpoints (VP2-3) contact the promoter and the enhancer and vice versa the enhancer contacts the *NIPBL* gene body (VP5), we might eventually observe here a tracking of the promoter that is still in contact with the enhancer along the *NIPBL* gene together with elongating RNA Pol II, as recently described by Lee *et al*. [70], although this remains to be tested in greater detail.

In summary, we have discovered very important features of the genetic context of the *NIPBL* gene and obtained functional insight on the regulation of the *NIPBL* gene expression. Although we did not observe a direct effect of the lncRNA for the *NIPBL* gene, we found that the transcription of the two genes is interconnected, suggesting a mechanism for the fine-tuning of *NIPBL* expression. With these experiments we have also demonstrated one possibility to boost the transcriptional activity of the *NIPBL* gene by interfering with the *NIPBL-AS1* lncRNA. This could be further explored for other genes with a similar arrangement of lncRNA and gene, and eventually also be used to manipulate gene expression in patient cells.

Moreover, we identified a distal enhancer controlling sense and antisense transcription at the bidirectional promoter of *NIPBL/NIPBL-AS1*. Given that even a modest reduction of *NIPBL* expression dramatically impacts on development, we suggest to include this non-coding genomic element into molecular diagnostics for CdLS.

## Materials and methods

### Cell culture

HEK293T cell line was cultured in DMEM supplemented with 0.2mM L-glutamine, 100 units/ml penicillin, 100 mg/ml streptomycin and 10% FCS and was grown at 37°C and 5% CO2.

HB2 cells (1-7HB2, a clonal derivative of the human mammary luminal epithelial cell line MTSV1-7, [71]) were cultured in DMEM supplemented with 0.2 mM L-glutamine, 100 units/ml penicillin, 100 mg/ml streptomycin, 10% FCS, 5 μg/ml hydroxycortisone and 10 μg/ml human insulin.

Lymphoblastoid cell lines derived from controls and Cornelia de Lange syndrome patients [20] were obtained from Ian Krantz (The Children’s Hospital of Philadelphia, Philadelphia, Pennsylvania, United States of America) and cultured in RPMI medium supplemented with 0.2mM L-glutamine, 100 units per ml penicillin, 100 mg per ml streptomycin, 20% FCS.

### Transcription analysis by reverse transcription (RT) and qPCR

Cells were harvested and total RNA was prepared using Trizol Reagent (Invitrogen). After chloroform extraction and isopropanol precipitation, pellets were dissolved in DEPC water. cDNA was generated by reverse transcription using oligo(dT)18 primer (Invitrogen), Superscript II Reverse Transcriptase (RT) (Invitrogen) and RNaseOUT Recombinant Ribonuclease Inhibitor (Invitrogen) according to the manufacturer’s instructions. The amounts of the different transcripts were compared by qPCR using SYBR Green and Platinum Taq Polymerase (Invitrogen) in CFX96 light cycler (BioRad) and specific primers. ΔΔCt method was used to calculate the fold change in gene expression using the housekeeping gene SNAPIN and the control sample for normalization.

### Design of Anti-Sense Oligonucleotides (ASO)

Anti-Sense Oligonucleotides (ASO) were designed followed the guidelines from Integrated DNA Technologies (IDT) (https://eu.idtdna.com/Scitools/Applications/AntiSense/Antisense.aspx?source=menu). Two ASO were designed against the 5’ and 3’ end of *NIBPL-AS1* and one were designed against a non-targeting sequence to use as control. All the ASOs were modified with a phosphorothioate (PS) linkages that confer nuclease resistance.

The following ASOs were used:

*ASO2 targeting the 5’ end of NIBP-AS1*: G*C*C* C*T*T* C*C*C* T*C*T* G*T*G* T*A*A* T*T*C
*ASO3 targeting the 3’ end of NIBP-AS1*: T*G*T* G*G*G* T*T*T* C*T*G* G*T*G* T*T*G* T*G*G
*Control ASO non targeting sequence*: A*T*A* T*T*T* C*C*A* C*G*C* C*A*G* C*C*A* G*A

The position of the phosphorothioate (PS) linkages is indicated as *.

### Depletion of *NIPBL-AS1* by ASO and transcription analysis by reverse transcription (RT) and qPCR

HB2 or HEK293T cells we transfected with 400 pmol of either ASO2, ASO3 or Control ASO (IDT) using Lipofectamine 2000 (Invitrogen) according to the manufacture’s instruction. Cells were harvested 48 hours after transfection and cDNA was prepared as described above. ΔΔCt method was used to calculate the fold change in gene expression using the housekeeping gene SNAPIN and the control sample for normalization.

### CAS9 constructs and guide RNA

The expression vectors for active Cas9 and catalytically inactive Cas9 were obtained from Addgene. The guide RNA were designed using the Cas9 design tool (http://cas9.cbi.pku.edu.cn/CasDesign) [72] and inserted in the gRNA Cloning Vector (Addgene #41824) [73] following the protocol deposited together with the vector. The sequences and positions of the guide RNA are listed in the S1 Table.

### Blocking of *NIPBL-AS1* or *NIPBL* transcription by CRISPR/CAS9 and transcription analysis by reverse transcription (RT) and qPCR

To block the transcription of *NIPBL-AS1*, two gRNAs (G1 and G2; Block I) targeting the 5’ end of *NIPBL-AS1* were used. To block the transcription of *NIPBL*, three gRNAs (G3, G4, G5; Block II) targeting the 5’ end of *NIPBL* were used. As controls we used either a combination of guide RNAs on *NIPBL* that are not effective for RNA PolII blocking (G6 and G7; Block III) or one guide RNA localizing outside of the locus (GC). The gRNA were designed using the web tool http://cas9.cbi.pku.edu.cn/CasDesign and cloned into a pCR-Blunt II-TOPO vector (Plasmid #41824 from AddGene) by Gibson Assembly (New England BioLabs) according to the manufacture’s instruction. HEK293T cells were transfected with the gRNAs vectors and the catalytically inactive Cas9 vector (dCas9) [74] (Plasmid #47948 from AddGene) using Lipofectamine 2000 (Invitrogen) according to the manufacture’s instruction. Cells were harvested 48 hours after transfection and cDNA was prepared as described above. ΔΔCt method was used to calculate the fold change in gene expression using the housekeeping gene SNAPIN and the Control sample for normalization.

### Chromosome conformation capture sequencing (3C-seq) and analysis

Chromosome conformation capture sequencing was performed as previously described in [55]. Briefly, cells were crosslinked with 1% (w/v) formaldehyde for 10 minutes and quenched with 120mM glycine. Crosslinked-cells were resuspended in lysis buffer (50mM Tris-HCl pH 8.0, 0.5% NP-40, 50mM NaCl and Complete protease inhibitor (Roche)) and subjected to enzymatic digestion using 400 units of BglII (Roche) or ApoI (New England Biolabs) for the higher resolution protocol. Digested chromatin was then diluted and ligated using 5 units of T4 DNA ligase (Promega) under conditions favoring intramolecular ligation events. After reversing the crosslink at 65°C over night, the digested and ligated chromatin was subjected to a second enzymatic digest using NlaIII (New England Biolabs) to produce smaller DNA fragment. The resulting digested DNA underwent a second ligation using 10 units of T4 DNA ligase (Promega) under conditions favoring self-ligation events that produce circular DNA molecules. The unknown DNA fragment, ligated to the fragment of interest (called viewpoint), was amplified by inverse-PCR using specific primer design in the outer part of the restriction site of the viewpoints, linked with the Illumina adapter sequences. The samples were then single-read sequenced using the Illumina Genome Analyzer II generating 76bp reads. The reads were trimmed to remove the Illumina adapter sequences and mapped against human genome (hg18 and hg19). Analysis was performed as previously described [1,75].

### Depletion of regulatory region R1 by CRISPR/Cas9

To deplete the candidate enhancers (R1_1 and R1_2) in region R1, three gRNAs (gRNA_1, gRNA2 and gRNA_3) targeting the region were designed and cloned as described above. To delete R1_1 (5 kb deletion in R1 region), HEK 293T cells were transfected with 2ug of gRNA2, gRNA3 and the vector coding for the catalytically active Cas9 (Plasmid #48139, AddGene) using Lipofectamine 2000 (Invitrogen) according to the manufacture’s instruction. To delete the entire R1 region including R1_1 and R1_2 (12 kb deletion), HEK293T cells were transfected with 2 μg of gRNA1, gRNA3 plasmids and the catalytically active Cas9. Cell were cultured in a puromycin-containing medium and single clones were picked and PCR analyzed for the presence of the corresponding deletion.

### Genotyping analysis of clones

Cells from different clones were harvested and lysed with lysis buffer (100mN Tris-HCl pH8.0, 5mM EDTA, 0.2% SDS, 50mM NaCl and proteinase K).

Genomic DNA was isolate using isopropanol and samples were analyzed by PCR. Primer sequences are listed in the Supplemental information.

### Transcription analysis by reverse transcription (RT) and qPCR of deletion clones

Clones positive for deletions D1 and D2 were grown, cells were harvested and cDNA was prepared as described above. HEK293T cells were used as control. ΔΔCt method was used to calculate the fold change in gene expression using the housekeeping gene SNAPIN and the Control sample for normalization.

### LncRNA fractionation in cytoplasmic and nuclear

Fractionation of cells was performed as described [76] with addition of RNAse inhibitors (RNAseout, Invitrogen) according to the manufacturer’s instructions. Preparation of RNA with from the fractions using Trizol and the cDNA preparation and subsequent qPCR analysis are described above.

### Enhancer reporter assay

To test whether the candidate enhancer has indeed an effect on the NIPBL promoter we cloned the luciferase gene under control of the NIPBL promoter (hg19, pos. chr5:36,875,913–36,876,915) together with the putative enhancer elements (R1_1—(hg 19)chr5:36743873–36745877, R1_2 –(hg 19)chr5:36738465–36742009) or a similar sized control region in the pGL4.10 vector (see scheme Fig 4A). Cloning primer sequences are available upon request.

HEK293 cells were transfected using FuGENE-HD (Promega, Madison, USA) with one of the following constructs: (1) empty pGL4.10 vector; (2) pGL4.10 vector containing R1_1 and NIPBL promoter; (3)(2) pGL4.10 vector containing R1_2 and NIPBL promoter; (4) pGL4.10 vector containing genomic DNA of the same size as the regulatory element. After 24 hours the cells were lysed and the activity of firefly and renilla luciferase determined with the Dual Luciferase Reporter Assay (Promega) in a TriStar2 LB Multidetection Microplate Reader (Berthold, Bad Wildbad, Germany). Genomic regions and primer sequences are given in the table. All measurements were verified in a minimum of three independent experiments and as triplicates in each experiment.

### ChIP-qPCR

The chromatin immunoprecipitation with anti-RNA PolII Ser5 and anti-PolII was performed as described [1].

### Pyrosequencing

A DNA fragment of interest was PCR-amplified with a biotinylated primer as described [77]. After denaturation, the biotinylated single-stranded PCR amplicon was hybridized with the sequencing primer, specific for the analyzed position. The allelic dosage was quantified on PyroMark Q24 instrument, with PyroMark Gold Q24 Reagent Kit (Qiagen), according to the manufacturer's instructions.

### Antibodies for western blotting

NIPBL—monoclonal rat anti-NIPBL, isoform A (long isoform) NP_597677 (Absea, China, 010702F01 clone KT54) and isoform B (short isoform) NP_056199 (Absea, China, 010516H10 clone KT55)

SMC2 –rabbit polyclonal anti-SMC2 [78]

Tubulin—mouse anti-tubulin (Sigma)

### Ethics Statement

There are no ethical issues. All cell lines used in this manuscript were previously published [20].

## Supporting information

S1 Fig Cellular localization of the *NIPBL-AS1* lncRNA and knockdown of the *NIPBL-AS1* in HB2 cells. A) Fractionation of the RNA in HEK293T cells into cytoplasmic and nucleoplasmic fraction and detection of *NIPBL-AS1* as well as the control lncRNAs *MALAT1* and *XIST* and the housekeeping genes *SNAPIN* and *NADH* with RT-qPCR (mean n = 3, error bars +/- s.d.).
B-C) Transcript levels of *NIPBL-AS1* (B) and *NIPBL* (C) after antisense oligonucleotide (ASO) knockdown of *NIPBL-AS1* in HB2 cells. Cells were transfected with either ASO2 or ASO3, targeting respectively the 5’ end or the 3’ end of *NIPBL-AS1* and one non-targeting control ASO (ASO C). Transcript levels were normalized against the control sample (ASO C) and the housekeeping *SNAPIN* (mean n = 6, error bars +/- s.d., p-values determined with t-Test).
(PDF)

S2 Fig Coverage of the *NIPBL* and *NIPBL-AS1* promoter with RNA PolII and RNA PolII Ser5 under the different CRIPSRi conditions. A) Overview of the *NIPBL-AS1* and the *NIPBL* promoter region together with ChIP-seq data for RNA polymerase II, CTCF, the H3K4me3 histone mark and DNase hypersensitive regions in HEK293 cells (ENCODE). The locations of the different guide RNAs used for the CRISPRi blocks (Block I, Block II and Block III) as well as the primer used for ChIP-qPCR are shown.
B-C) Enrichment of Ser5-phosphorylated initiating RNA polymerase (Ser 5, panel B) and general RNA Pol II (PolII, panel C) when transcription of *NIPBL-AS1* is blocked (Block I).
D-E) Enrichment of Ser5-phosphorylated initiating RNA polymerase (Ser 5, panel D) and general RNA Pol II (PolII, panel E) when transcription of *NIPBL* is blocked (Block II).
The position of the guide RNA furthest into the gene body together with the ChIP primer are highlighted with blue boxes–left side: Block I primer AS3 in the *NIPBL-AS1* gene—right side: Block II primer AS7 in the *NIPBL* gene. ChIP-qPCR results are expressed as fold enrichment relative to the target region AS3 on each control (Block III) [79] (average n = 3 experiments, error bars +/- s.d., p-values determined with paired two-tailed t-Test).
(PDF)

S3 Fig Long range interaction of the *NIPBL/NIPBL-AS1* promoter in HB2 cells. A) Long-range chromosomal interactions of the region covering the *NIPBL* and *NIPBL-AS1* promoter (VP1) detected by chromosome conformation capture (3C-seq) in the breast epithelial cell line HB2 using an BglII digest. The positions of the viewpoints are highlighted in yellow. Note that two viewpoints (VP2 and VP3) were positioned further into the *NIPBL* gene to validate the long-range interaction of the promoter (P) into the *NIPBL* gene body.
B) Validation of interactions between the promoter region (P) (NIPBL_VP4, blue track) and two candidate regions R1 and R2 carrying enhancer marks (R1—VP5, green track and R2—VP6, red track) using the more frequently cutting enzyme ApoI in HB2 cells.
C) CTCF ChIP sequencing track from HEK293 cells (ENCODE) and DNAse hypersensitivity. The orientations of the CTCF motifs as determined with JASPAR are shown below the track (red triangle–forward orientation, green triangle–reverse orientation). The CTCF sites involved in the promoter-enhancer interaction are indicated with yellow triangles above the track.
D) Histone modification profiles—H2A.z, H3K4me1, H3K4me2 and H3K4me3—of six different cell lines (G312878, K562, HeLa-S3, HEMEC, HSMM and HUVEC, available from ENCODE) are displayed as density graph in which black represents areas with the highest enrichment of the ChIP-sequencing signals. *NIPBL* and *NIPBL-AS1* promoter region (P) and distal intragenic regions (R1 and R2) detected by 3C-sequencing analysis are highlighted with blue boxes.
(PDF)

S4 Fig Interactions between the *NIPBL* promoter/*NIPBL-AS1* and distal enhancers are conserved between different human cell lines and in part also in mouse. Hi-C interactions maps at 5 kb resolution from seven different human cell lines [59] (maps generated with http://promoter.bx.psu.edu/hi-c/view.php) (A-G) and in the CH12 mouse cell line (H). Interactions between the *NIPBL* promoter/*NIPBL-AS1* and the potential enhancer in R1 are indicated by dashed lines. When available in ENCODE ChIP-seq signals for CTCF and different histone marks are shown. In GM12878 cells (A) also region R2 is shown and the interaction of R2 with the *NIPBL* promoter that is unique for this cell line is indicated with an arrow. Note that the potential enhancer in mouse cells (H) is positioned closer to the *Slc1a3* gene than in human cells.
(PDF)

S5 Fig Deletion of the potential enhancer using CRISPR/Cas9. A) Location of the gRNAs (gRNA_1, gRNA_2 and gRNA_3) used to delete the potential enhancers R1_1 and R1_2. The ENCODE data for CTCF in HEK293 cell and histone marks (H2A.z, H3K4me1, H3K4me2 and H3K4me3) derived from six different cell lines (G312878, K562, HeLa-S3, HEMEC, HSMM and HUVEC) are shown to support that these regions are potential enhancers. Note that the combination of gRNA_2 and gRNA_3 will delete one CTCF binding site and the combination of gRNA_1 and gRNA_3 will delete two CTCF binding sites.
(B-C) Schematic overview of the two different conditions used to create (B) a partial deletion of 5 kb (D1, gRNA2+gRNA3) or (C) a full deletion of 12 kb (D2, gRNA1 +gRNA3). The primers used for genotyping of the clones and the respective PCR product sizes are shown.
(D-H) Analysis of CRISPR edited clones with deletions D1 and D2. Genomic DNA of the clones was analysed with PCR primers specific for the deletions (for primer positions see B and C) and PCR products analysed on agarose gels. (D) PCR products in unedited HEK293T cells (Control). Note that primers P4-P8 give only in unedited cells a product of correct size. (E-H) Genotyping of clones obtained in two rounds of CRIPSR targeting. Clones D1_89 and D2_35 were obtained in the first round. In the second round four clones were obtained for D1 and three for D2, clones D1_63 and D2_103 are shown as examples. (E+F) Genotyping of D1 clones using one primer designed for a product unique for the D1 deletion (P2, 815bp product) and primers designed to detect the intact genomic region (P6-P8). (G+H) Genotyping of D2 clones using one primer designed for a product unique for the D2 deletion (P3, 927bp) and primer designed to detect the intact genomic region (P4-P8).
The expected product sizes are indicated on the agarose gel pictures in (E) and (G). The PCR products missing due to the deletions are indicated with boxes. The asterisks (*) indicate side-products of the PCR primers that become more prominent in the absence of the original templates.
(PDF)

S6 Fig *NIPBL-AS1* and *NIPBL* mRNA levels in the clones deleted for R1_1 (D1) and both R1_1 and R1_2 (D2). Transcript levels of the individual clones and the HEK293T cells used for genome edition were determined with two primers for *NIPBL* (#42 and #138) and one for *NIPBL-AS1* (#132) (mean n = 3 of cDNA preparations from the clones, error bars +/- s.d., p-values determined with t-Test).
(PDF)

S7 Fig mRNA levels of *NIPBL* regulated genes in the enhancer deletion clones. Transcript levels of the genes *BBX*, *GLCCI1* and *ZNF695* that were described as dysregulated genes in CdLS [20] and previously confirmed as NIPBL-dependent genes with NIPBL binding sites at the promoter [8] were analysed in all enhancer deletion clones R1_1 (D1) and both R1_1 and R1_2 (D2) (mean n = 3 from different cDNA preparations, error bars +/- s.d., p-values determined with t-Test).
(PDF)

S8 Fig Levels of *NIPBL* and *NIPBL-AS1* transcripts in CdLS patient and control LCLs. A) Details of control and CdLS patients lymphoblastoid cell lines (LCLs) used for analysing *NIPBL* and *NIPBL-AS1* transcripts. The lines were previously described [8,20].
B) Transcript levels of *NIPBL* and *NIPBL-AS1* in four controls and three CdLS patients. Two primer pairs for *NIPBL* and one for *NIPBL-AS1* were used. Transcript levels were normalized against the housekeeping gene *NADH*. Note that transcript levels are reduced by only 30–40% in CdLS patients but the *NIPBL-AS1* transcription is hardly affected.
C) The contribution of intact and mutated allele to the total RNA was determined in PT1 and PT3 by pyrosequencing to estimate the efficiency of nonsense-mediated decay. To visualize that the intact and mutant allele are transcribed at similar level nonsense mediated decay was blocked with cycloheximide.
(PDF)

S1 Table Guide RNA used in the different experiments. (PDF)

S2 Table Primer used to detect the deletions generated by CRISPR/Cas9. (PDF)

S3 Table 3C–seq viewpoint primer pairs. (PDF)

S4 Table Primer used for qPCR analysis of transcripts and for ChIP-qPCR analysis. (PDF)
