# Supplementary material for: Regulation of the cohesin-loading factor NIPBL: Role of the lncRNA NIPBL-AS1 and identification of a distal enhancer element
Source: PLoS Genet. 2017 Dec 20;13(12):e1007137. doi: 10.1371/journal.pgen.1007137 (PMC5754091; doi:10.1371/journal.pgen.1007137)

FIGURE S1

A Cellular localization of the NIPBL-AS1 noncoding RNA

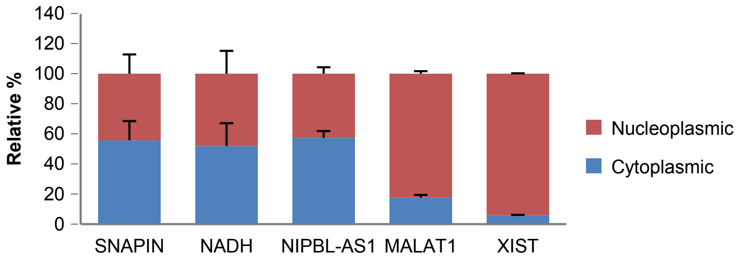

NIPBL-AS1 and NIPBL mRNA levels after ASO knockdown in HB2 cells:

B **NIPBL-AS1** (#132)

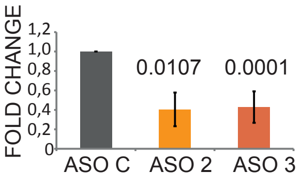

C **NIPBL** (#138) **NIPBL** (#42)

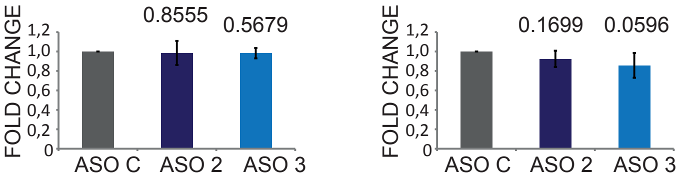

Supplement: S1 Fig — A) Fractionation of the RNA in HEK293T cells into cytoplasmic and nucleoplasmic fraction and detection of NIPBL-AS1 as well as the control lncRNAs MALAT1 and XIST and the housekeeping genes SNAPIN and NADH with RT-qPCR (mean n = 3, error bars +/- s.d.). B-C) Transcript levels of NIPBL-AS1 (B) and NIPBL (C) after antisense oligonucleotide (ASO) knockdown of NIPBL-AS1 in HB2 cells. Cells were transfected with either ASO2 or ASO3, targeting respectively the 5’ end or the 3’ end of NIPBL-AS1 and one non-targeting control ASO (ASO C). Transcript levels were normalized against the control sample (ASO C) and the housekeeping SNAPIN (mean n = 6, error bars +/- s.d., p-values determined with t-Test). (PDF) [file pgen.1007137.s001.pdf]
