# Supplementary figures and images for: Regulation of the cohesin-loading factor NIPBL: Role of the lncRNA NIPBL-AS1 and identification of a distal enhancer element
Source: PLoS Genet. 2017 Dec 20;13(12):e1007137. doi: 10.1371/journal.pgen.1007137 (PMC5754091; doi:10.1371/journal.pgen.1007137)

FIGURE S2

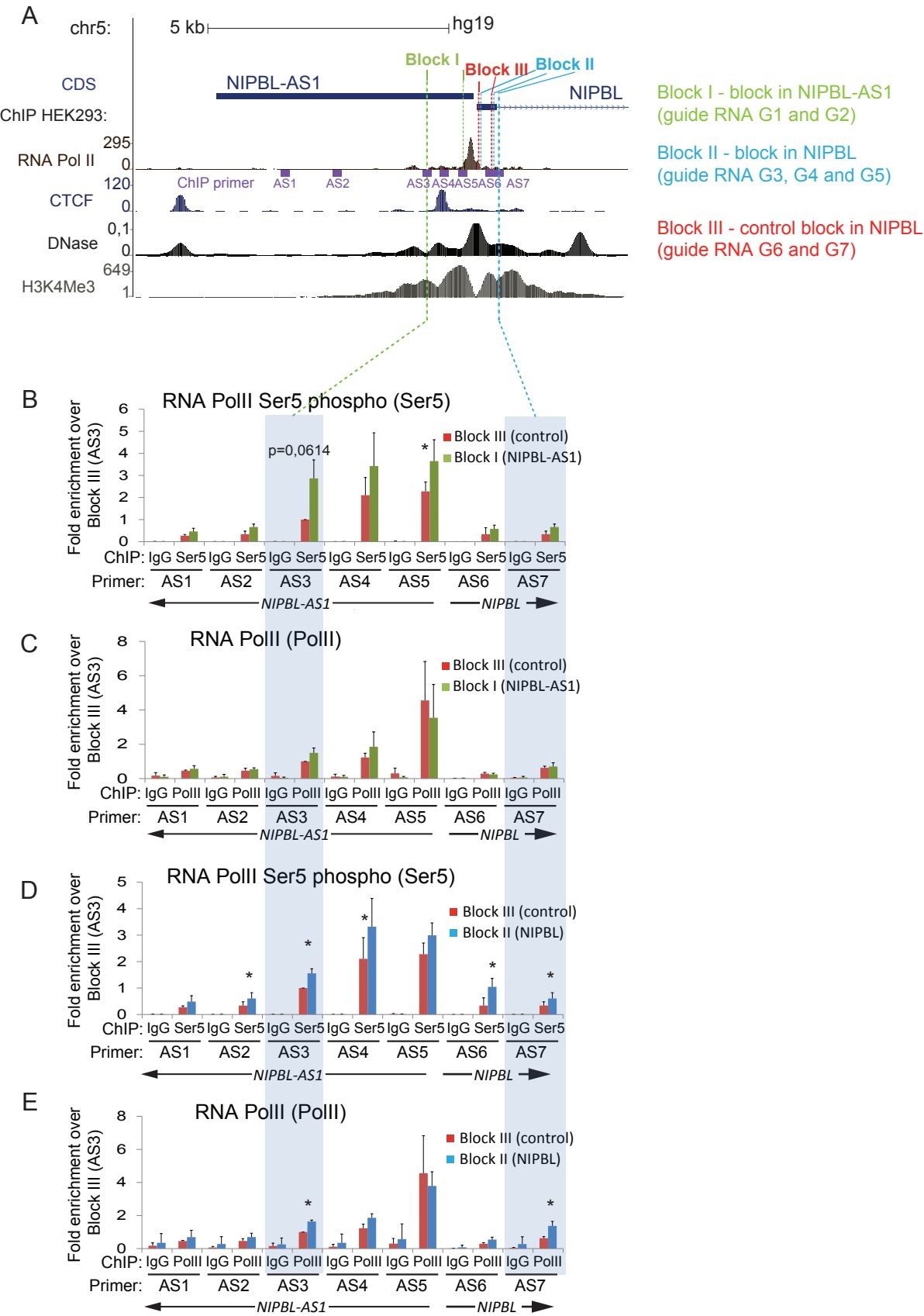

Paired two-tailed T-test using three replicates: \* p< 0,05

Supplement: S2 Fig — A) Overview of the NIPBL-AS1 and the NIPBL promoter region together with ChIP-seq data for RNA polymerase II, CTCF, the H3K4me3 histone mark and DNase hypersensitive regions in HEK293 cells (ENCODE). The locations of the different guide RNAs used for the CRISPRi blocks (Block I, Block II and Block III) as well as the primer used for ChIP-qPCR are shown. B-C) Enrichment of Ser5-phosphorylated initiating RNA polymerase (Ser 5, panel B) and general RNA Pol II (PolII, panel C) when transcription of NIPBL-AS1 is blocked (Block I). D-E) Enrichment of Ser5-phosphorylated initiating RNA polymerase (Ser 5, panel D) and general RNA Pol II (PolII, panel E) when transcription of NIPBL is blocked (Block II). The position of the guide RNA furthest into the gene body together with the ChIP primer are highlighted with blue boxes–left side: Block I primer AS3 in the NIPBL-AS1 gene—right side: Block II primer AS7 in the NIPBL gene. ChIP-qPCR results are expressed as fold enrichment relative to the target region AS3 on each control (Block III) [79] (average n = 3 experiments, error bars +/- s.d., p-values determined with paired two-tailed t-Test). (PDF) [file pgen.1007137.s002.pdf]

FIGURE S3:

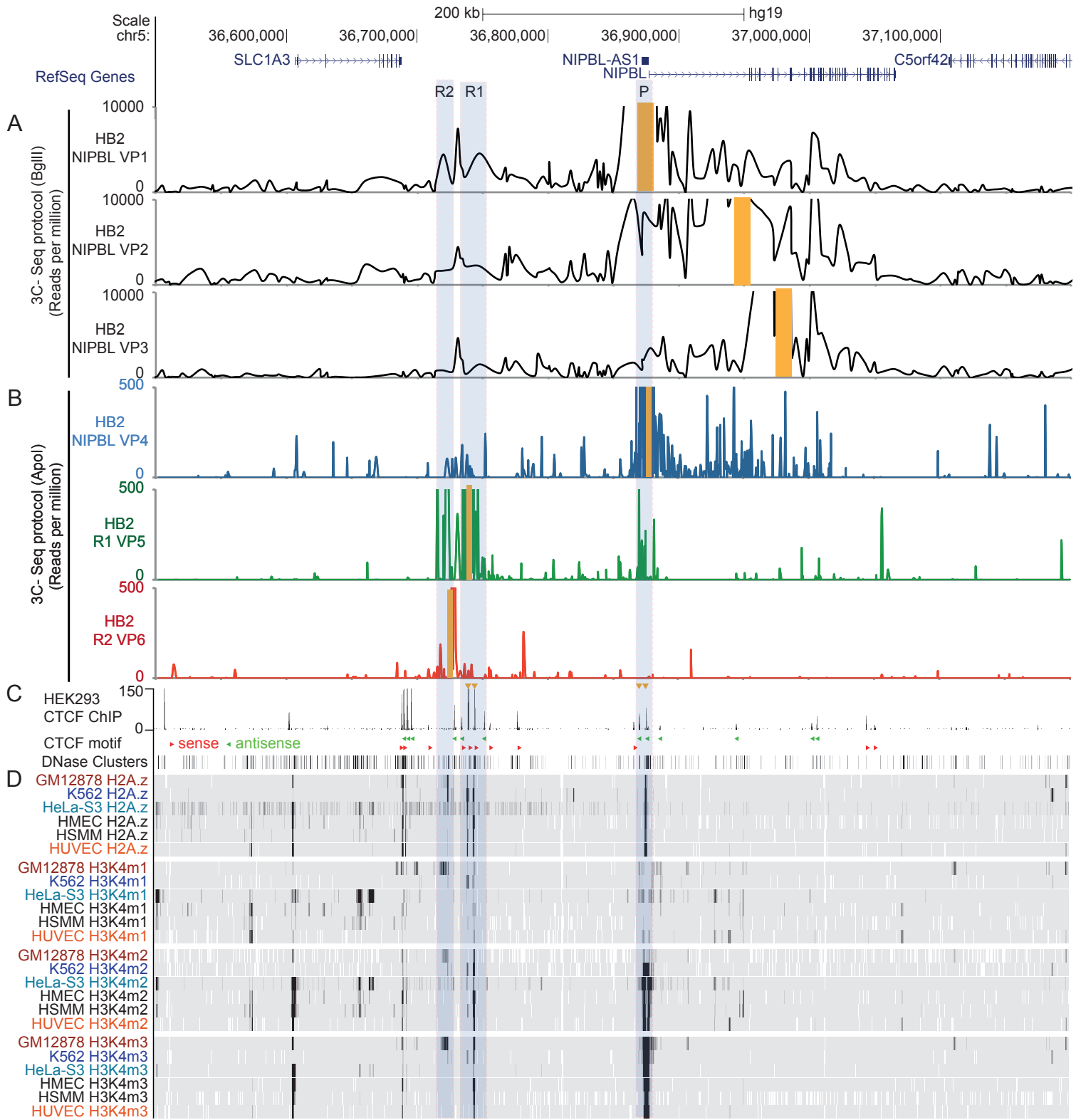

Supplement: S3 Fig — A) Long-range chromosomal interactions of the region covering the NIPBL and NIPBL-AS1 promoter (VP1) detected by chromosome conformation capture (3C-seq) in the breast epithelial cell line HB2 using an BglII digest. The positions of the viewpoints are highlighted in yellow. Note that two viewpoints (VP2 and VP3) were positioned further into the NIPBL gene to validate the long-range interaction of the promoter (P) into the NIPBL gene body. B) Validation of interactions between the promoter region (P) (NIPBL_VP4, blue track) and two candidate regions R1 and R2 carrying enhancer marks (R1—VP5, green track and R2—VP6, red track) using the more frequently cutting enzyme ApoI in HB2 cells. C) CTCF ChIP sequencing track from HEK293 cells (ENCODE) and DNAse hypersensitivity. The orientations of the CTCF motifs as determined with JASPAR are shown below the track (red triangle–forward orientation, green triangle–reverse orientation). The CTCF sites involved in the promoter-enhancer interaction are indicated with yellow triangles above the track. D) Histone modification profiles—H2A.z, H3K4me1, H3K4me2 and H3K4me3—of six different cell lines (G312878, K562, HeLa-S3, HEMEC, HSMM and HUVEC, available from ENCODE) are displayed as density graph in which black represents areas with the highest enrichment of the ChIP-sequencing signals. NIPBL and NIPBL-AS1 promoter region (P) and distal intragenic regions (R1 and R2) detected by 3C-sequencing analysis are highlighted with blue boxes. (PDF) [file pgen.1007137.s003.pdf]

FIGURE S5

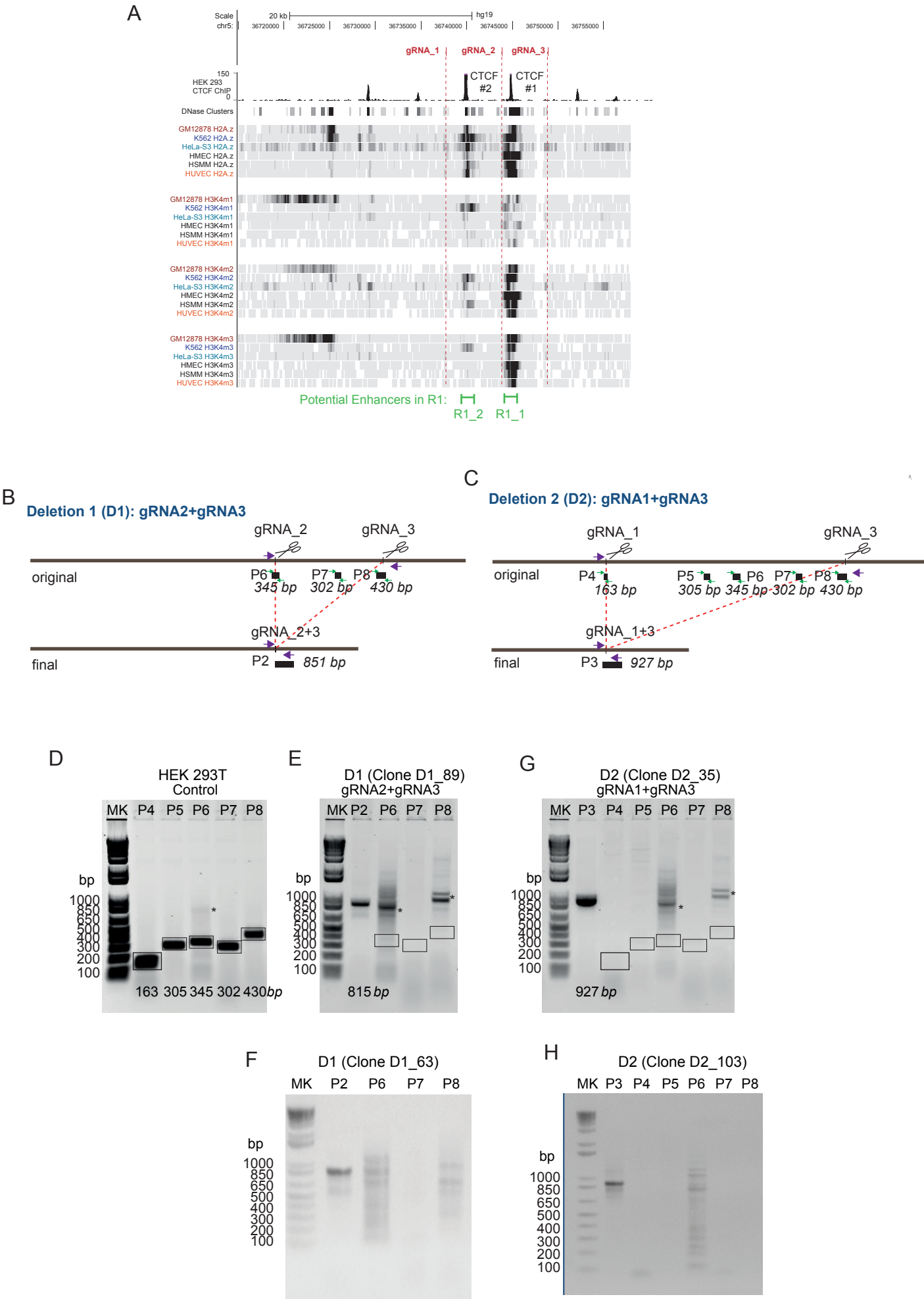

Supplement: S5 Fig — A) Location of the gRNAs (gRNA_1, gRNA_2 and gRNA_3) used to delete the potential enhancers R1_1 and R1_2. The ENCODE data for CTCF in HEK293 cell and histone marks (H2A.z, H3K4me1, H3K4me2 and H3K4me3) derived from six different cell lines (G312878, K562, HeLa-S3, HEMEC, HSMM and HUVEC) are shown to support that these regions are potential enhancers. Note that the combination of gRNA_2 and gRNA_3 will delete one CTCF binding site and the combination of gRNA_1 and gRNA_3 will delete two CTCF binding sites. (B-C) Schematic overview of the two different conditions used to create (B) a partial deletion of 5 kb (D1, gRNA2+gRNA3) or (C) a full deletion of 12 kb (D2, gRNA1 +gRNA3). The primers used for genotyping of the clones and the respective PCR product sizes are shown. (D-H) Analysis of CRISPR edited clones with deletions D1 and D2. Genomic DNA of the clones was analysed with PCR primers specific for the deletions (for primer positions see B and C) and PCR products analysed on agarose gels. (D) PCR products in unedited HEK293T cells (Control). Note that primers P4-P8 give only in unedited cells a product of correct size. (E-H) Genotyping of clones obtained in two rounds of CRIPSR targeting. Clones D1_89 and D2_35 were obtained in the first round. In the second round four clones were obtained for D1 and three for D2, clones D1_63 and D2_103 are shown as examples. (E+F) Genotyping of D1 clones using one primer designed for a product unique for the D1 deletion (P2, 815bp product) and primers designed to detect the intact genomic region (P6-P8). (G+H) Genotyping of D2 clones using one primer designed for a product unique for the D2 deletion (P3, 927bp) and primer designed to detect the intact genomic region (P4-P8). The expected product sizes are indicated on the agarose gel pictures in (E) and (G). The PCR products missing due to the deletions are indicated with boxes. The asterisks (*) indicate side-products of the PCR primers that become more prominent in the absence [file pgen.1007137.s005.pdf]

FIGURE S6:

NIPBL-AS1 and NIPBL mRNA levels in the clones deleted for R1\_1 (D1) and both R1\_1 and R1\_2 (D2)

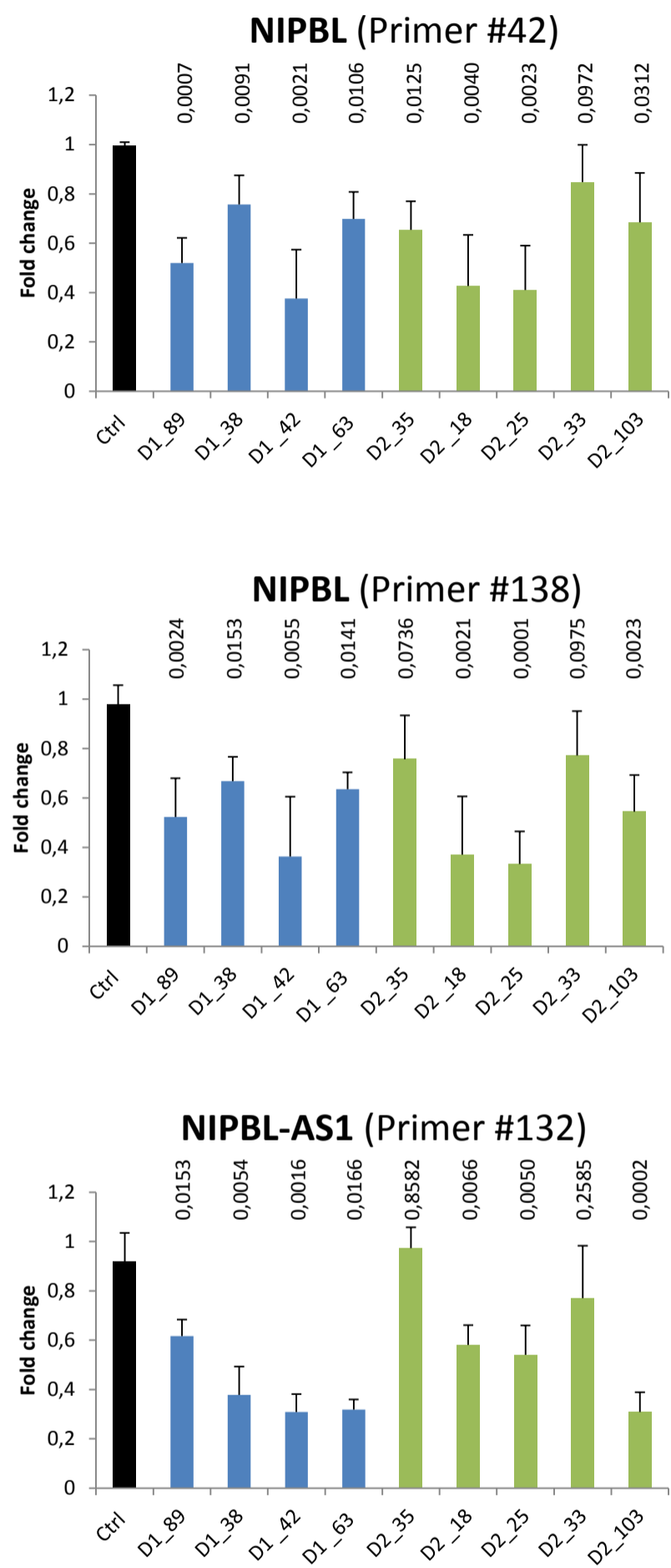

Supplement: S6 Fig — Transcript levels of the individual clones and the HEK293T cells used for genome edition were determined with two primers for NIPBL (#42 and #138) and one for NIPBL-AS1 (#132) (mean n = 3 of cDNA preparations from the clones, error bars +/- s.d., p-values determined with t-Test). (PDF) [file pgen.1007137.s006.pdf]

FIGURE S7:

mRNA levels of NIPBL regulated genes in the clones deleted for R1\_1 (D1) and both R1\_1 and R1\_2 (D2)

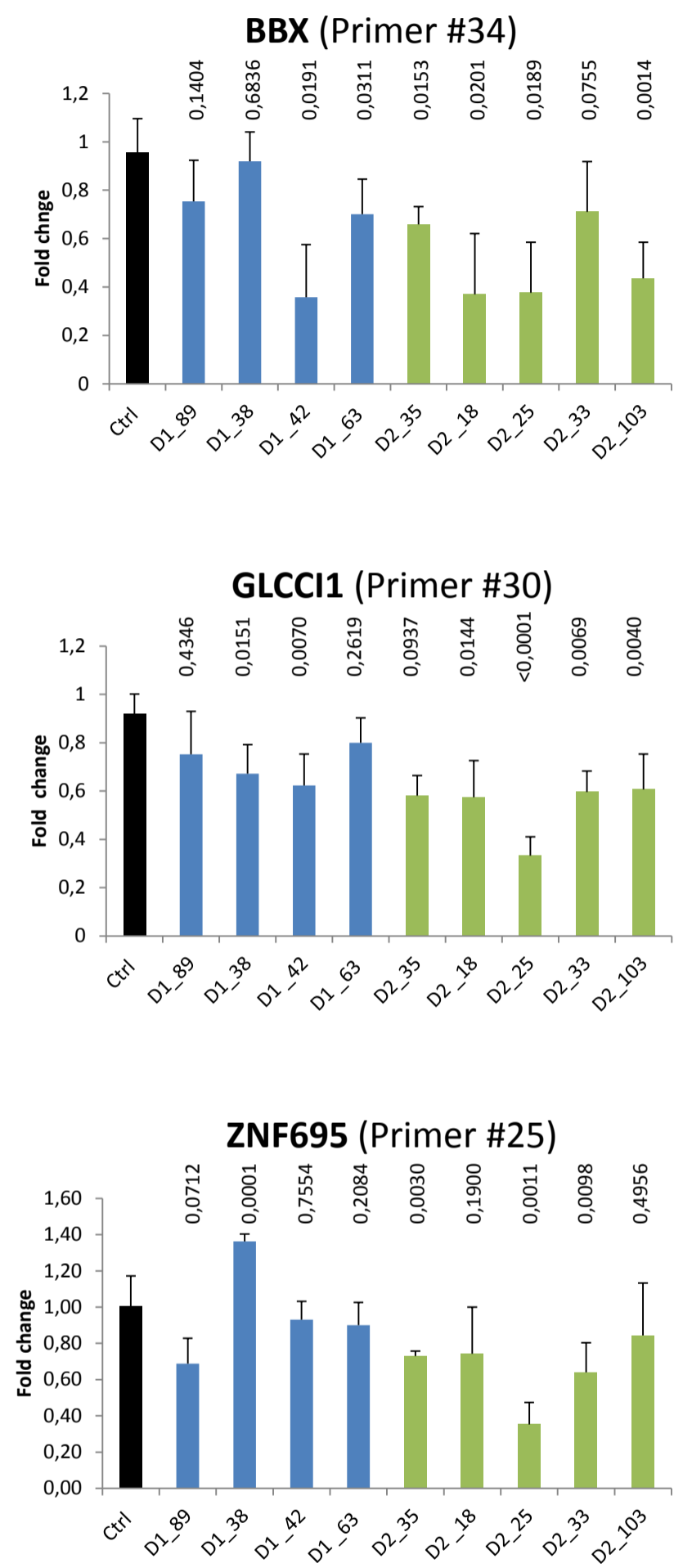

Supplement: S7 Fig — Transcript levels of the genes BBX, GLCCI1 and ZNF695 that were described as dysregulated genes in CdLS [20] and previously confirmed as NIPBL-dependent genes with NIPBL binding sites at the promoter [8] were analysed in all enhancer deletion clones R1_1 (D1) and both R1_1 and R1_2 (D2) (mean n = 3 from different cDNA preparations, error bars +/- s.d., p-values determined with t-Test). (PDF) [file pgen.1007137.s007.pdf]

FIGURE S8

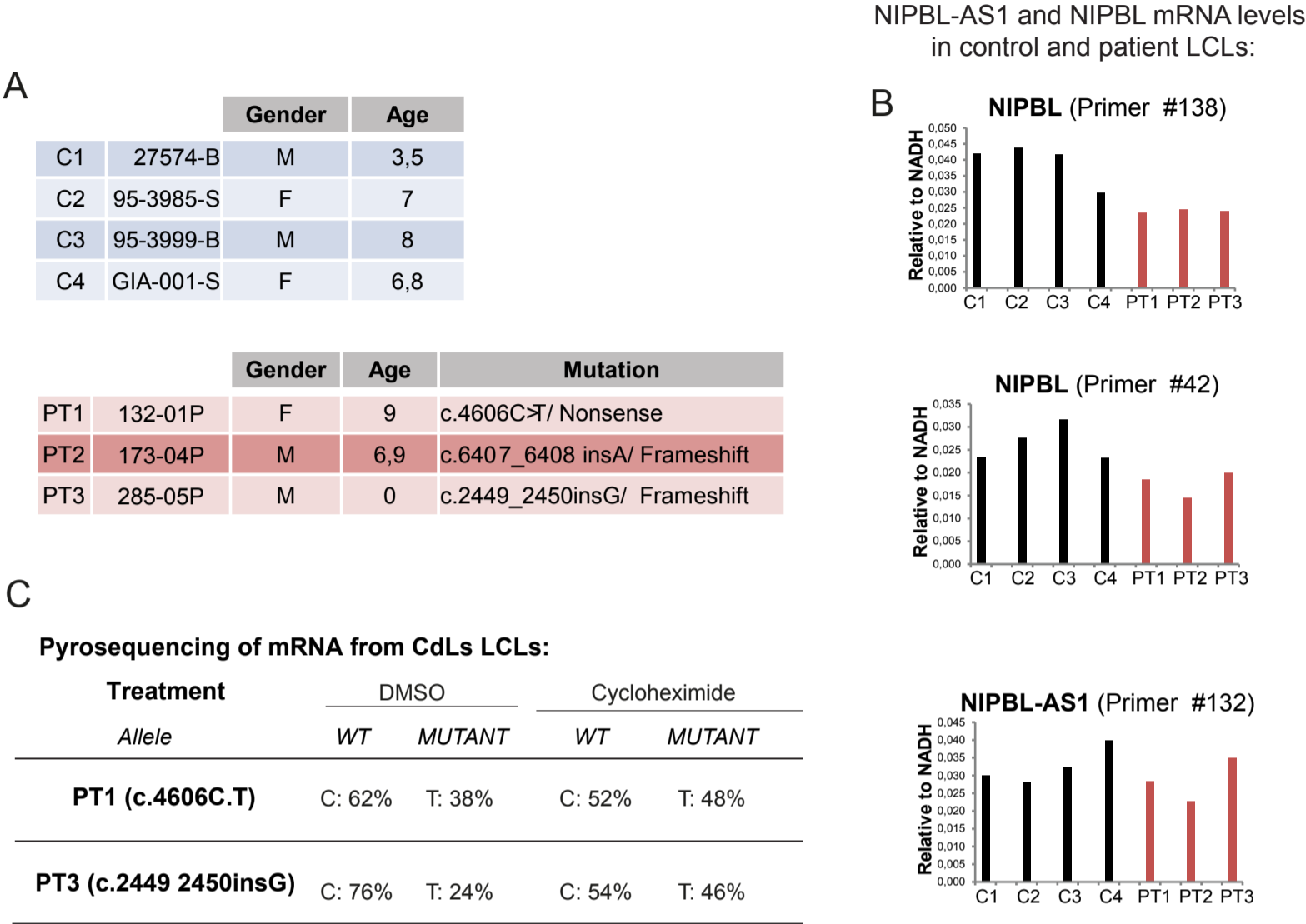

Supplement: S8 Fig — A) Details of control and CdLS patients lymphoblastoid cell lines (LCLs) used for analysing NIPBL and NIPBL-AS1 transcripts. The lines were previously described [8,20]. B) Transcript levels of NIPBL and NIPBL-AS1 in four controls and three CdLS patients. Two primer pairs for NIPBL and one for NIPBL-AS1 were used. Transcript levels were normalized against the housekeeping gene NADH. Note that transcript levels are reduced by only 30–40% in CdLS patients but the NIPBL-AS1 transcription is hardly affected. C) The contribution of intact and mutated allele to the total RNA was determined in PT1 and PT3 by pyrosequencing to estimate the efficiency of nonsense-mediated decay. To visualize that the intact and mutant allele are transcribed at similar level nonsense mediated decay was blocked with cycloheximide. (PDF) [file pgen.1007137.s008.pdf]
