# Supplementary material for: Regulation of the cohesin-loading factor NIPBL: Role of the lncRNA NIPBL-AS1 and identification of a distal enhancer element
Source: PLoS Genet. 2017 Dec 20;13(12):e1007137. doi: 10.1371/journal.pgen.1007137 (PMC5754091; doi:10.1371/journal.pgen.1007137)
Supplement: S1 Table — (PDF) [file pgen.1007137.s009.pdf]

S2 Table

Guide RNA used in the different experiments

Experiment	gRNA Name	Target Region	Position (hg19)
**Control gRNA**	GC_F	gAAAGACATCTGCCTTGACAG	chr5:36759180-36759199
	GC_R	CTGTCAAGGCAGATGTCTTc	
**gRNAs for blocking NIPBL-AS1 transcription**	G1_F	gATAGAGAACGGTGGAACAA	chr5:36875822-36875844
	G1_R	TTGTTCCACCGTTCTCTATc	
	G2_F	gTCCAGGAAAATAAAGACAG	chr5:36876573-36876595
	G2_R	CTGTCTTTATTTTCCTGGAc	
**gRNAs for blocking NIPBL transcription (designed on minus strand-->target plus strand)**	G3_F	gGACGCTGAGGACCTGGCGC	chr5:36877325-36877344
	G3_R	GCGCCAGGTCCTCAGCGTCc	
	G4_F	gGTGGCGAGAGACTATAATC	chr5:36877218-36877237
	G4_R	GATTATAGTCTCTCGCCACc	
	G5_F	gGTGAGTCGGTACCGACGGA	chr5:36876949-36876968
	G5_R	TCCGTCGGTACCGACTCACc	
**gRNAs for blocking NIPBL transcription (designed on plus strand-->target minus strand)**	G6_F	gCGACAGGAGAATTGGTTCC	chr5:36877003-36877022
	G6_R	GGAACCAATTCTCCTGTCGc	
	G7_F	AACGATTTGTCTTCTCGGC	chr5:36877120-36877139
	G7_R	GCCGAGAAGACAAATCGTTc	
**gRNAs for R1 region deletion**	gRNA1_F	gAGGCAGCCAGGCAGTTTCT	chr5:36737782-36737804
	gRNA1_R	GAAACTGCCTGGCTGCCTc	
	gRNA2_F	gGCTGCGGATACACCTTGCA	chr5:36743873-36743895
	gRNA2_R	TGCAAGGTGTATCCGCAGCc	
	gRNA3_F	gTTCCTTCTACGCTCACTGA	chr5:36748879-36748901
	gRNA3_R	TCAGTGAGCGTAGAAGGAAc	
