# Supplementary material for: Regulation of the cohesin-loading factor NIPBL: Role of the lncRNA NIPBL-AS1 and identification of a distal enhancer element
Source: PLoS Genet. 2017 Dec 20;13(12):e1007137. doi: 10.1371/journal.pgen.1007137 (PMC5754091; doi:10.1371/journal.pgen.1007137)
Supplement: S2 Table — (PDF) [file pgen.1007137.s010.pdf]

S2 Table

Primer used to detect the deletions generated by CRISPR/Cas9

Primer to detect deletions in R1	Target region	Position (hg19)
P2_F	TGTGGCTTGGCCTAATTG	chr5:36743828-36749685
P2_R	CAGAGGCTGGTTCTCTTG	
P3_F	AACGCGAGCCTCTTCTCTTCC	chr5:36737654-36749678
P3_R	TGGTTCTCTTGGTCCAGGAGG	
P4_F	CCCAACACCTGTTCCTCTTAC	chr5:36737678-36737840
P4_R	CCTGGGACTGACATTTCTGTTC	
P5_F	CTCAAGGGCAGACACAATG	chr5:36742317-36742621
P5_R	GGCACTTACTCTGTGAAAGG	
P6_F	GCCCAAATAGCACTGCAGAC	chr5:36743671-36744015
P6_R	GAAAGTGGCAGAGCTGTGAG	
P7_F	GGTACATGGCTCCCATTTGAAC	chr5:36746584-36746885
P7_R	AGTGCCTAGGTAAGGAACCTTG	
P8_F	ACTACATGCCAGGCACAATC	chr5:36748530-36748959
P8_R	TGGCATTTCTGGCCTAACTC	
