# Supplementary material for: Regulation of the cohesin-loading factor NIPBL: Role of the lncRNA NIPBL-AS1 and identification of a distal enhancer element
Source: PLoS Genet. 2017 Dec 20;13(12):e1007137. doi: 10.1371/journal.pgen.1007137 (PMC5754091; doi:10.1371/journal.pgen.1007137)
Supplement: S3 Table — (PDF) [file pgen.1007137.s011.pdf]

S3 Table

3C-seq viewpoint primer pairs

Viewpoint	Primer Name	Sequence	Position
VP1	BglII_F<br>NlaIII_ R	ATTAGATTAACAGATTCTATGTAAG<br>CCTCCTTGCTTCAAGAAC	chr5:36873783-36873807
VP2	BglII_F<br>NlaIII_R	TTTGGTCAAACGTTGAG<br>ATGGAAACTTTTTGAGATGTA	chr5:36944718-36944738
VP3	BglII_F<br>NlaII_R	CTCGTATCTGCTTCATATGG<br>CTCAAACCTGGACTTAAATTC	chr5:36973150-36973170
VP4	ApoI_F<br>NlaIII_R	CGCCGATTCGCCCAGGTA<br>TCTTCCGTCTCCTCAGTGCG	chr5:36877168-36877283
VP5	NlaIII_F<br>ApoI_R	GAGCACTGACTTGGTCTTC<br>CAGTGGATGGGGAATGAAT	chr5:36739739-36740347
VP6	NlaIII_F<br>ApoI_R	ACGTGGCAGGCTTCAT<br>CAGATCCGTTTGCCTGGAAT	chr5:36725938-36726330
