# Supplementary material for: Regulation of the cohesin-loading factor NIPBL: Role of the lncRNA NIPBL-AS1 and identification of a distal enhancer element
Source: PLoS Genet. 2017 Dec 20;13(12):e1007137. doi: 10.1371/journal.pgen.1007137 (PMC5754091; doi:10.1371/journal.pgen.1007137)
Supplement: S4 Fig — Hi-C interactions maps at 5 kb resolution from seven different human cell lines [59] (maps generated with http://promoter.bx.psu.edu/hi-c/view.php) (A-G) and in the CH12 mouse cell line (H). Interactions between the NIPBL promoter/NIPBL-AS1 and the potential enhancer in R1 are indicated by dashed lines. When available in ENCODE ChIP-seq signals for CTCF and different histone marks are shown. In GM12878 cells (A) also region R2 is shown and the interaction of R2 with the NIPBL promoter that is unique for this cell line is indicated with an arrow. Note that the potential enhancer in mouse cells (H) is positioned closer to the Slc1a3 gene than in human cells. (PDF) [file pgen.1007137.s004.pdf]

FIGURE S4

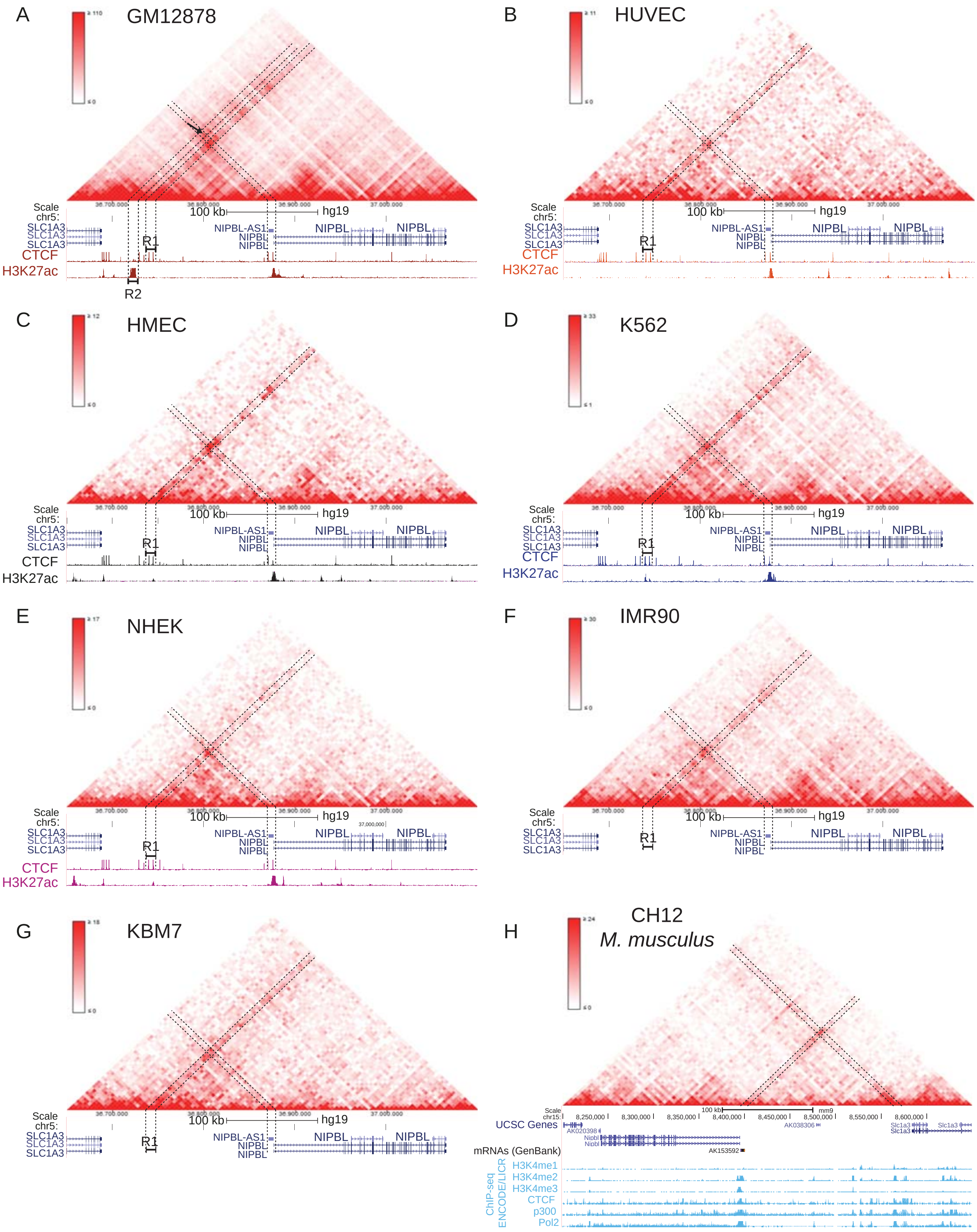

A
GM12878
B
HUVEC
C
HMEC
D
K562
E
NHEK
F
IMR90
G
KBM7
H
CH12
M. musculus
Scale
chr5:
100 kb
hg19
SLC1A3
NIPBL-AS1
NIPBL
R1
R2
CTCF
H3K27ac
chr15:
mm9
UCSC Genes
mRNAs (GenBank)
Nipbl
Slc1a3
ChIP-seq
ENCODE/LICR
H3K4me1
H3K4me2
H3K4me3
p300
Pol2
