# Supplementary material for: Regulation of the cohesin-loading factor NIPBL: Role of the lncRNA NIPBL-AS1 and identification of a distal enhancer element
Source: PLoS Genet. 2017 Dec 20;13(12):e1007137. doi: 10.1371/journal.pgen.1007137 (PMC5754091; doi:10.1371/journal.pgen.1007137)
Supplement: S4 Table — (PDF) [file pgen.1007137.s012.pdf]

S4 Table

Primer used for qPCR analysis of transcripts

Primer Nr.	orientation	Sequence
132*	forward	TTTCCTAGTGGACCTACATTTG
	reverse	CCCATGTTTATTATTGTGCTGTATC
138*	forward	GTCGCAGCAAGAACAAAC
	reverse	GCCTCCTGTGGGTAAGAATC
42*	forward	GGAGTGACATGGCTAATTCC
	reverse	TCATCAGGGTCCAGATGTTC
TC25	forward	AGCGGAGTTTGTATAGGG
	reverse	GTTTGGCTGTCTTCTCTG
TC30	forward	AGGCGAACCTCCTCTTTGG
	reverse	GACGCAGAACGCTGATGTG
TC34	forward	GGAATCCTGCTGCATCAC
	reverse	TCGTCCTCCTCTTCTTCC
NADH	forward	ACTGGCTACTGCGTACATCC
	reverse	AGATGCGCCTATCTCTTTCC
SNAPIN	forward	AGCTCGACTCTCACGTACAC
	reverse	GCCGGGCATTAAGTAGCTTC
NIPBL nascent	forward	GTCGCAGCAAGAACAAAC
	reverse	GATGAAGTGGTAATAATGAGA
MALAT1	forward	GATAATCAGACCACAGGTTTACAG
	reverse	AAAGAGTAACTACCAGCCATTTCTCCA
XIST	forward	ACATGCCTGGCACTCTAGCA
	reverse	AAACATGGAAATGGGTAAGACACA

*The positions of these primers is indicated in Figure 1A.

Primer used for ChIP-qPCR analysis

Primer Nr.	orientation	Sequence
AS1	forward	AGCGCCTAATAAGTCCTC
	reverse	CAGCTGCTACAGTCAATC
AS2	forward	CCTGACCTCAGCTTTCATTG
	reverse	GTACCAGGCACGCTAATTC
AS3	forward	GAATTTCGTCCCGGTTGAG
	reverse	CTGGACAAGGCTGGAATAC
AS4	forward	GCCTTTATCCTGACCGTTAG
	reverse	TCGGCTGGATTGAATTGG
AS5	forward	GCTCACGACCACCACCGAACTC
	reverse	CTCAGGGCGGCTGTGGATGTTG
S1	forward	CGTGGTCGGGTGTTTGTGAGTG
	reverse	ACTATAATCCGGGCCGGGAG
S2	forward	GCCTCCCCTTGGATTCAGAC
	reverse	CACCAGAGAGGAGCGAACTC

The positions of all primers is indicated in Figure S2A.
